# Functional Module Connectivity Map (FMCM): A Framework for Searching Repurposed Drug Compounds for Systems Treatment of Cancer and an Application to Colorectal Adenocarcinoma

**DOI:** 10.1371/journal.pone.0086299

**Published:** 2014-01-27

**Authors:** Feng-Hsiang Chung, Yun-Ru Chiang, Ai-Lun Tseng, Yung-Chuan Sung, Jean Lu, Min-Chang Huang, Nianhan Ma, Hoong-Chien Lee

**Affiliations:** 1 Institute of Systems Biology and Bioinformatics, National Central University, Zhongli, Taiwan; 2 Center for Dynamical Biomarkers and Translational Medicine, National Central University, Zhongli, Taiwan; 3 Division of Hematology and Oncology, Cathay General Hospital, Taipei, Taiwan; 4 Institute of Biomedical Science, Academia Sinica, Nangang, Taipei, Taiwan; 5 Department of Physics, Chung Yuan Christian University, Zhongli, Taiwan; 6 Cathay Medical Research Institute, Cathay General Hospital, Taipei, Taiwan; 7 Physics Division, National Center for Theoretical Sciences, Hsinchu, Taiwan; Huazhong University of Science and Technology, China

## Abstract

Drug repurposing has become an increasingly attractive approach to drug development owing to the ever-growing cost of new drug discovery and frequent withdrawal of successful drugs caused by side effect issues. Here, we devised Functional Module Connectivity Map (FMCM) for the discovery of repurposed drug compounds for systems treatment of complex diseases, and applied it to colorectal adenocarcinoma. FMCM used multiple functional gene modules to query the Connectivity Map (CMap). The functional modules were built around hub genes identified, through a gene selection by trend-of-disease-progression (GSToP) procedure, from condition-specific gene-gene interaction networks constructed from sets of cohort gene expression microarrays. The candidate drug compounds were restricted to drugs exhibiting predicted minimal intracellular harmful side effects. We tested FMCM against the common practice of selecting drugs using a genomic signature represented by a single set of individual genes to query CMap (IGCM), and found FMCM to have higher robustness, accuracy, specificity, and reproducibility in identifying known anti-cancer agents. Among the 46 drug candidates selected by FMCM for colorectal adenocarcinoma treatment, 65% had literature support for association with anti-cancer activities, and 60% of the drugs predicted to have harmful effects on cancer had been reported to be associated with carcinogens/immune suppressors. Compounds were formed from the selected drug candidates where in each compound the component drugs collectively were beneficial to all the functional modules while no single component drug was harmful to any of the modules. In cell viability tests, we identified four candidate drugs: GW-8510, etacrynic acid, ginkgolide A, and 6-azathymine, as having high inhibitory activities against cancer cells. Through microarray experiments we confirmed the novel functional links predicted for three candidate drugs: phenoxybenzamine (broad effects), GW-8510 (cell cycle), and imipenem (immune system). We believe FMCM can be usefully applied to repurposed drug discovery for systems treatment of other types of cancer and other complex diseases.

## Introduction

An important goal for biomedical research is to understand the underling genetic mechanisms of human diseases and discover therapeutic drugs for the diseases. Drug discovery is expensive; the average research and development (R&D) cost in the past 15 years for developing a new drug exceeds 1 billion US dollars [1]. Anti-cancer agents are especially costly [2]. The standard R&D procedure includes compound identification, toxicity tests in cell and animal models, safety evaluation on early clinical trials, and efficacy in late phase trials. The very high failure rate has led to a crisis known as innovation gap in new drug discovery [3]. The crisis is further compounded by the withdrawal of many previously thought successful drugs, mostly through issues related to harmful side effects [4]–[6]. Such issues may be a corollary of the prevailing method for new drug discovery, which is to find specific biomolecules as targets, typically membrane receptors [7]. However, a biological target may regulate more than one biological pathway, only one of which may be disease related. If this is the case, then altering the function of a biological target by a drug can lead to unintended results of disruption of healthy pathways [8].

The strategy of finding specific biological targets for drug treatment may have also contributed to the disappointing progress made in the last 40 years in reducing the overall mortality rates for most types of cancer [9]. This is partly because cancer is a disease involving the dysfunction of multiple parallel pathways controlling many fundamental processes [10]. Cancer cells accumulate multiple genetic mutations that equip it with a of myriad of survival and death-avoiding capabilities: for inducing angiogenesis; to maintain proliferative signalling; to escape suicidal apoptotic programs; for enabling replicative immortality; and to activate invasion and metastasis [11]. Evidences are emerging that the pathology of cancer is more a consequence of small abnormalities on many genes, than a major abnormality on a single gene [12], [13], and that drug compounds acting on multiple targets may be a more effective treatment strategy than a single drug on a single target [14]. In short, cancer is a systems disease and ought to be dealt with by a systems treatment [15].

Here, we present a computational drug-screening procedure that addresses the issues raised above. Our program has two main aims: to surmount the innovation gap through drug repurposing, and to find drug compounds for a systems treatment of cancer. Drug repurposing is the search for novel indications for already approved drugs [1], [16]. Because an approved dug has already been optimized for safety and efficacy for its originally designed indication, its route for approval for a novel indication may be significantly shorter and likely far less costly than that for a new drug.

Recently the computational screening drugs for repurposing has been greatly facilitated by the advent of the Connectivity Map (CMap), a comprehensive and continuously updated database of the genomic profiles of many existing drugs [17]. CMap provides a platform for utilizing a pattern-matching strategy to determine the similarity, or the opposite, in genomic signatures among diseases, functional gene sets, and drugs. It has been employed in many studies for discovering repurposing drugs against common diseases, including diabetes and Alzheimer's disease [17], and for treating solid tumours, including those associated with colon cancer [18], breast cancer [19], and lung adenocarcinoma [20].

The basic concept of CMap-based repurposing drugs discovery studies is the identification of disease associated genomic signatures that reversely correlate with perturbation in genomic signature associated with the administration of molecules or drugs [17], [21], [22]. In these studies, the essence of the protocol – the individual-gene CMap approach (IGMP) – for identifying drugs for treating a specific disease is straightforward: find a set of differentially expression genes (DEGs) obtained by, say, comparing two sets – controls and patients – of gene expression microarrays, score the match between the DEG set and genomic profiles of drugs given by CMap, and rank the drugs by score. Candidate drugs are those with the highest scores. Because it draws on the entire genomic information of the patients and of the drug, one may view this approach as an attempt at systems treatment. However, it suffers from being too crude an approach. In particular it makes no specific reference to any of the many altered states of biological functions associated with the disease. By not paying attention to individual biological functions, a “best” drug could very well be a compromise, chosen for having strong beneficial effects on a subset of functions at the expense of being harmful to some other functions.

Another study that utilizes variable gene signatures to screen repurposed drugs has successfully identified many heterogeneous Food and Drug Administration (FDA) approved drug candidates for breast, myelogenous leukemia, and prostate cancer [23]. This method typically yields a long list of heterogeneous drug candidates without providing details that may help in differentiating the drugs, details such as how a drug differently impact the multi-functionalities of (a specific) cancer. Other more sophisticated methods based on computational network models have been developed to identify novel therapeutic targets for the purpose of treating regulatory cellular networks [24], [25]. The effectiveness of these approaches, which aim to elevate the relative activity of certain cell regulatory networks, and base their predictions on elaborate models optimally tuned to fit existing temporal and spatial data, may be restricted by the limited existing knowledge on networks and parameters describing protein activities.

Here, we present a novel analytical framework, called Functional Module Connectivity Map (FMCM), for the discovery of drug compounds for systems cancer treatment. We constructed condition-specific function-function networks (FFNs) and applied a gene-selection-by-trend-of-progression procedure (GSToP) [26] to identify complexly connected and highly expressed hub genes in the FFNs. We then used functional modules constructed around the hub genes to query CMap for the discovery of ontology-specific repurposing drugs, and further screened the drugs by requiring that they exhibit minimum intracellular harmful side effects. Relative to the standard IGCM protocol, FMCM was more robust in its drug selection and it more consistently predicted higher hit rates (∼65%) on effective drugs against early tumorigenesis in colorectal cancer. When checked against known drug indications in Therapeutic Target Database (TTD), FMCM showed significantly higher accuracy and lower false positive rates on the discovery of the anti-cancer agents than IGCM, except for the immune system. Our viability tests on eight of the candidate drugs showed three, GW-8510, ginkgolide A, and 6-azathymine, represented high inhibitory activities against the survival of cancer cell lines with specific concentrations and administration durations. Follow-up microarray experiments confirmed that both the CMap and our datasets showed consistent results on three independent drugs – phenoxybenzamine (broad effects), GW-8510 (cell cycle), and imipenem (immune system). These results demonstrated the effectiveness of FMCM, and suggested its potential for formulating repurposed drug regimes with minimum harmful side effects in cancer patients.

## Materials and Methods

### Data sources

Gene expression data for 32 patients with sporadic colorectal polyps (adenoma) and corresponding adjacent normal mucosa from the same individuals were obtained from Gene Expression Omnibus (GEO) database (accession number: GSE8671) [27]. We extracted 30,047 protein entries and 39,194 protein-protein interactions (PPIs) from the Human Protein Reference Database (HPRD) [28] and used Gene Ontology (GO) [29] for functional information.

### External database

We used the Connectivity Map database (CMap) build 02 [17], with 6,100 treatment expression profiles representing 1,309 drugs (and compounds), to compute enrichment scores (ES) of gene set against drugs.

For reference on drug indication we used L01 class, antineoplastic agents, Anatomical Therapeutic Chemical (ATC) Classification System, World Health Organization (WHO) (http://www.whocc.no/).

We extracted information on known therapeutic protein targets, relevant diseases or cancers, and corresponding drugs (787 drugs; 60% of CMap datasets) from the Therapeutic Target Database (TTD: http://bidd.nus.edu.sg/group/ttd/) [30]. In addition, we queried key words on searching engines to define relative therapeutic drugs on cancer treatment.

We downloaded the annotated 4,884 gene-sets from the Molecular Signatures Database (MSigDB: http://www.broadinstitute.org/gsea/msigdb/index.jsp) [31]. The gene-sets are of four types: C2: curated gene-sets from known pathways, online databases, and knowledge of domain experts; C3: motif gene-sets based on conservative cis-regulatory motifs from human, mouse, rat, and dog genomes; C4: computational gene-sets determined by co-expression neighbourhoods centered on 380 cancer-related genes; C5: gene-ontology gene-sets collected from the same GO annotations of genes. Gene symbols in each gene-set were combined and converted into HG-U133A Affymetrix ID according to the updated annotation file at the website http://www.affymetrix.com/estore/.

### Gene selection by individual gene analysis (IGA) and Individual gene connectivity map (IGCM)

Differentially expressed genes (DEGs) were selected using the Significance Analysis of Microarrays algorithm (SAM) [32]. Unless otherwise stated, threshold values for false discovery rate (FDR) <0.05 and fold change (FC) >2 were used. Enrichment score (ES) matching gene set to drug was computed through CMap [17].

### Beneficial and harmful drugs

Given a gene set, a drug was designated beneficial or harmful if the ES is <−0.5 or >0.3. For drugs to be designated beneficial a randomization *p*-value<0.005 was required, unless otherwise stated.

### Construction of gene-gene interaction network (GGIN) and function-function interaction network (FFN)

For a given condition – control (Nor) or adenoma patient (Ade) – and a Pearson *p*-value (see below) threshold *p*_0_, we included a pair of genes in the GGIN if: (1) the *p*-value for the pair was not greater than *p*_0_; (2) the protein pair encoded by the gene pair was linked in the PPI data. For a given set of *n* microarrays, a Pearson's correlation coefficient (PCC) between a pair of genes was calculated using the two sets of *n* intensities of the pair. Each PPC is assigned a Pearson *p*-value based on permutation tests and *t*-statistics. Genes in each type-specific GGIN were assigned to over-represented biological functions, called functional modules, through Gene Ontology term association [29]. Enrichment analyses based on conditional hypergeometric test [33] were made using the R package GOstats downloaded from the Bioconductor website. Each GGIN was reduced to function-function network (FFN) using functional modules as nodes.

### GSToP and the functional module connectivity map (FMCM) framework

**Figure 1:**
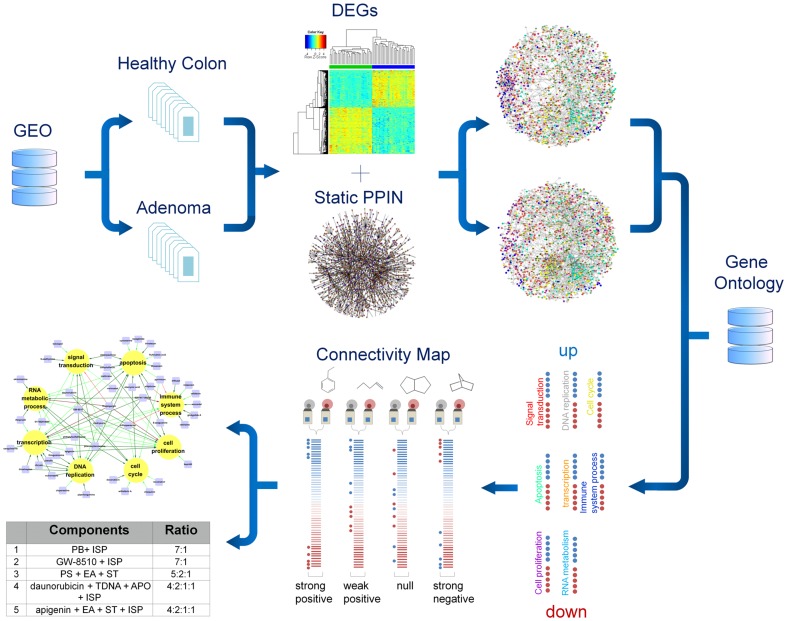
Flowchart of methodology.

The FMCM framework for selecting therapeutic drug compound consisted of two segments, selection of functional modules of predicted cancer genes based on the GSToP procedure [26] (steps 1–5 below), and multiple queries, one for each functional module, of the CMap for drug identification (steps 6–8). Steps in the selection procedure (Figure 1) were: (1) Construct Nor and Ade GGINs and FFNs using threshold Pearson *p*-value = 0.001. (2) SAM. Identify DEGs for Ade vs. Nor using thresholds FDR <0.01 and FC >2. (3) GSToP. Assign a gene as a cancer gene if: (a) it appears in at least the Ade or Nor GGIN; (b) its degrees and clustering coefficients increase (decrease) along the sequence. (4) Take overlap of SAM and GSToP lists. (5) Cancer genes (including up-regulated and down-regulated genes) form functional modules having GO terms used for the FFNs. (6) Beneficial and harmful drug lists. Use functional modules separately to query drugs in the CMap [17] to obtain for each function two lists respectively for predicted beneficial (ES <−0.5) and harmful (ES >0.3) drugs (see above for requirement on randomization *p*-value). (7) Function-drug association map (FDAM). Use the drug lists to construct a map with two kinds of nodes, function module and drug, and two kinds of function-drug links, beneficial and harmful. Include in FDAM only drugs that have at least one beneficial link. (8) Construct from FDAM all predicted drug compounds, where a compound is minimum set of purely beneficial drugs that covered all functions.

### Accuracy, specificity, and reproducibility in performance tests

Positives NB and negatives NA were drugs predicted to be beneficial and harmful, respectively; true positives TP and false negatives FN were known anti-tumor agents predicted to be beneficial and harmful, respectively; true negatives is TN = NA–FN, and false positive is FP = NB–TP. Accuracy was defined as (TP+TN)/(NB+NA), and specificity as TN/(FP+TN).

For reproducibility a drug prediction procedure (FMCM or IGCM) was repeated 10 times, each time working on a set of 40 randomly chosen microarrays, 20 each from controls and patients, and reproducibility was measured over the 10×9/2 = 45 pairs of results. For each pair reproducibility was (the size of) the intersection of the two sets of selected drugs divided by the geometric mean of the two sets.

### Cell cultures and reagents

Human colon cancer cell lines (HCT116, RKO, SW403, and SW620), and breast cancer cell lines (MCF7) were obtained from ATCC (American Type Culture Collection, Manassas, VA) and maintained as suggested by ATCC. The growth media for all cell lines were supplemented with 10% fetal bovine serum (FBS), 50 units/ml of penicillin and streptomycin, and incubated at 37°C with 5% carbon dioxide. In experiments, cells were treated with ethanol, water or DMSO as corresponding vehicle control. Phenoxybenzamine, GW-8510, etacrynic acid, ginkgolide A, triflusal, imipenem, 6-azathymine were purchased from Santa cruz (CA). Phthalylsulfathiazole was purchased from Sigma (St. Louis, MO). Phenoxybenzamine, phthalylsulfathiazole, etacrynic acid, ginkgolide were dissolved in ethanol. Imipenem was dissolved in water. The remaining drugs were dissolved in Dimethyl sulfoxide (DMSO).

### Cell Proliferation assay

Proliferation activities of five cell lines – colon cancer, HCT116, RKO, SW403, and SW620, and breast cancer, MCF7 – were monitored by Alamar Blue (Molecular Probes, Invitrogen Corporation), an oxidation-reduction reagent, and determined by measuring the reduction of resazurin (blue, non-fluorescent) to resorufin (red, highly fluorescent). Cells were seeded in 96-well cultured plates and, following the study design of the CMap [17], treated with single drugs with concentration of 0, 0.1, 1,10, 30 µM for 5 days, then assayed for proliferation activities. One-tenth volume of alamar blue reagent was added and plates were incubated at 37°C for 2–3 hours. Cell viability was determined by measuring fluorescence with excitation at 550 nm and emission at 590 nm on Synergy HT (BioTek Instruments, Winooski, VT). Cell survival was calculated as relative value of the difference between the reductions of Alamar Blue in treated versus controls.

### RNA extraction and microarrays

Cells were seeded in 100 mm dishes and treated with drugs. After drug treatment for 6 hours, total cellular RNA was isolated using mirVana miRNA Isolation Kit (Ambion, Austin, TX) according to the manufacturer's instructions. 250 ng of total RNA was used for microarray experiments. Extracted RNA was labelled with GeneChip® 3′ IVT Express Kit (Affymetrix, Santa Clara, CA, USA) and hybridized onto Affymetrix GeneChip® Prime View Human Gene Expression Array. This array contained approximately 530,000 probes covering more than 36,000 transcripts and variants. Raw images were analysed by Affymetrix GeneChip® Operating Software. We performed microarray analysis of the effect of imipenem and phenoxybenzamine (PB) (treated and non-treated) on HCT116 and MCF7, and GW-8510 on HCT116 under the U219 (primeview) platform, all in duplicates. Treatment dosages and duration times were the same as in [17].

### Microarray experiments and analysis by IGA and gene-set approach (GSA)

Genome-wide gene expression profiles from drug-perturbed tumour cells evaluated by the Affymetrix GeneChip® Prime View platform were analyzed in R environment (version 2.15.1). Two cell lines, HCT116 and MCF7, were treated with three drugs, GW-8510, phenoxybenzamine (PB), and imipenem, with the same dosages (10 uM, 11.8 uM, 13.4 uM, respectively) and time (6 hours after overnight culture) as in [17]. The microarray profiles were compared with ten profiles from the CMap for MCF7 treated with the three drugs. Gene expression intensities were normalized by Robust Multi-array Average (RMA) [34]. In the IGA approach DEGs were identified by one-way ANOVA using the eBayes function in the limma package [35]. In a gene-set approach (GSA), given a list of ranked differential gene expressions, we used GSEA [31] to convert the 4,884 annotated gene sets in MSigDB [31] to a list of 4,884 ranked ESs, then applied one-way ANOVA to find differential gene sets. In IGA (or GSA) a gene (or a gene-set) with false discovery rate (FDR) less than 0.01 was considered significant and selected for two-way hierarchical clustering of the microarray set. GO terms for overrepresented gene (or gene-set) clusters in the IGA (or GSA) heatmap were determined using DAVID [36].

## Results

### Function-function networks

**Figure 2:**
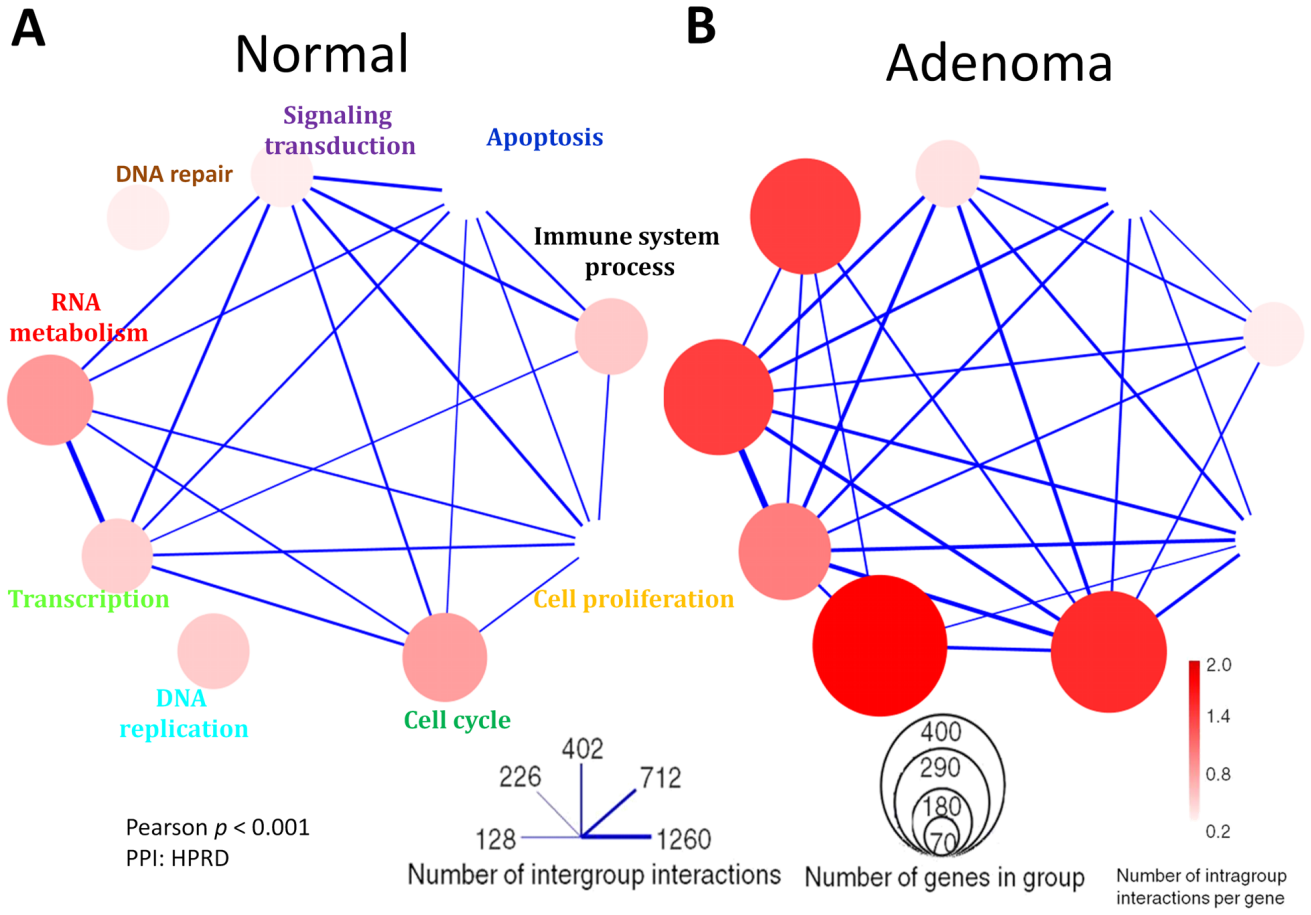
Function-function networks for colorectal adenoma. Condition specific function-function networks (FFNs) were generated from gene-gene networks (GGINs), shown in Figure S2, by reduction. Nodes in an FFN are functional modules (FMs), which are gene sets in the corresponding GGIN forming over-represented Gene Ontology terms. FMs containing less than 70 genes are not shown. The diameter of a node scales with the logarithm of the number of genes in the node. The color shade of a node indicates the number of intra-node gene-gene interactions per gene. The thickness of the edge indicates the number of inter-node gene-gene interactions.

High quality of microarray data was indicated by the clean separation of control (from 32 normal tissues) and sample (32 patients) in Principle Component Analysis (Figure S1A). The 2,164 DEGs selected by SAM (with thresholds FDR <0.01 and FC >2) correctly classified the controls and sample in hierarchical clustering (Figure S1B). Clustering results were not sensitive to moderate variations in threshold values (not shown). Gene-gene interaction networks (GGINs) were constructed with a threshold of Pearson *p*<0.001 from the control and adenoma cohort microarray data (Figure S2). The adenoma GGIN has 6.4% more genes (1,792 vs. 1,684) and 32% more links (2,656 vs. 2,017) than the control GGIN. The difference between the two cases became evident when the GGINs were reduced to function-function networks (FFNs) having functional modules as nodes (Figure 2, Table S1). Cell cycle, DNA replication, and DNA repair functional modules were much larger in the adenoma FFN and exhibited much high levels of intra-function activity. There were also more inter-module activities in adenoma than in control. In a noted exception, the inter-module activity between immune system process and cell proliferation was weaker in adenoma than in control.

### Repurposed therapeutic drugs selected by IGCM

**Figure 3:**
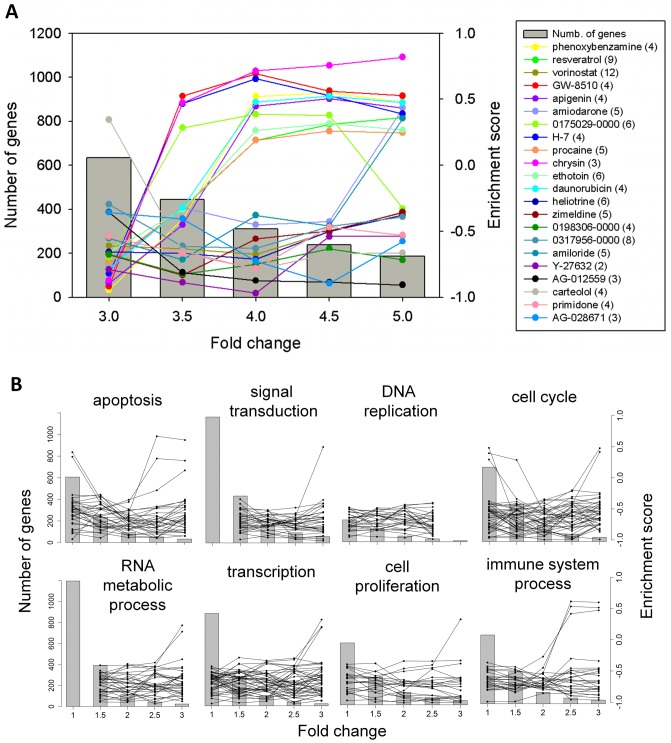
Enrichment score versus fold-change for CMap drugs. Enrichment score (ES) was obtained by querying the CMap with gene set (size indicated by vertical bar) determined using varying fold-change (FC) threshold. A drug is considered beneficial for the treatment for colorectal adenoma if ES <−0.5, harmful if ES >0.3, and neutral otherwise. (A) Screening by IGCM procedure. Querying gene set was complete set of differentially expressed genes (DEGs) identified from gene expression arrays of colorectal adenoma cohort (versus control) using the SAM algorithm with fixed FDR <0.01. (B) Screening by FMCM procedure. Querying gene sets were functional modules obtained by partition of over-represented Gene Ontology terms in GSToP filtered DEGs.

CMap gives enrichment scores (ES) to gene lists not longer than 1,000 entries. We complied with this constraint (i.e., restricting the size of the DEGs) by requiring FDR <0.01. Five DEG lists with FC thresholds of 3.0 to 5.0 with 0.5 intervals were generated and their ES's for the 1,308 drugs (or small compounds) were obtained by querying CMap. The list of beneficial (i.e., anti-adenoma) drugs was sensitive to (the threshold value of) FC, with the size of the list decreasing with increasing FC (Figure 3A). The number of beneficial validated drugs decreased with increasing FC (Figure S3). According to TTD, many known therapeutic anti-cancer drugs, such as chrysin (pink, TTD id: DNC004715), GW-8510 (red, TTD id: DNC004631), daunorubicin (cyan, TTD id: DAP000788), apigenin (light purple, TTD id: DNC004714), resveratrol (yellow green, TTD id: DNC001205), coincidentally all changed from beneficial at FC = 3 to harmful at FC = 3.5 (Figure 3A). At FC = 5.0, the most stringent threshold that we used mostly, AG-012559 was the only beneficial drug under permutation *p*<0.005 (Figure S3).

### Repurposed therapeutic drugs selected by FMCM

In the FMCM program, genes selected in each functional module (Table S2) were used separately to query CMap, yielding separate functional specific drug lists. Each functional module was the union of the control and adenoma functional modules given by the respective FFNs, filtered by the GSToP procedure (see Methods). In FMCM the gene size of the module had a much stronger dependence on the value of FC than IGCM (Figure 3). In IGCM, size of DEG dropped from just above 600 to 200 when the value of FC threshold was raised from 3 to 5. In FMCM module gene size dropped from about 600 to about 30 as (the threshold) FC was raised from 1 to 3, and became too small for CMap application when FC was raised above 3. Even so, within the respective range of FC used, FMCM provided a much more stable and robust environment for drug screening by CMap than IGCM; in FMCM the character of selected drugs (i.e., beneficial or harmful) changed very little (21 out of 256, Figure 3B) while in IGCM changed occurred to 54.5% of selected drugs (12 out of 22, Figure 3A).

### Function-drug association map (FDAM) and therapeutic drug compounds

**Figure 4:**
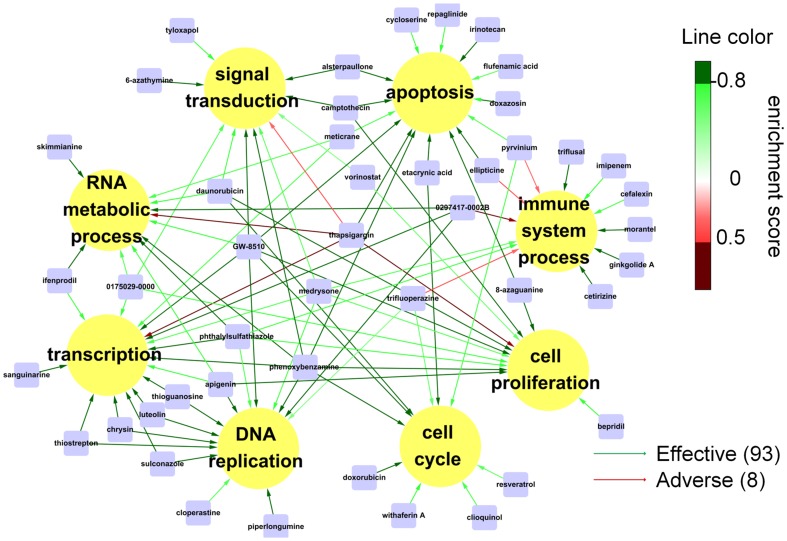
Function-drug association map (FDAM) for colorectal adenoma. Nodes in the map are functional modules (FMs; gene sets) and drugs obtained by querying CMap using individual FMs. Drug-function links indicate beneficial (green) or harmful (red). Only drugs beneficial to at least one FM are included.

Purely beneficial and harmful drugs (see Material and Methods) that were beneficial to at least one FM were identified in the FMCM program (FC >2) and used to construct FDAM. The 46 drugs in the FDAM (Table 1) were much more numerous than the corresponding list found in traditional IGCM approach (which had 22 drugs). Thirty of the 46, or 65%, have either been studied individually as anti-tumour agents or have been certified to have preventive effects against a broad range of cancers (Table 1 and Table S3). The five drugs, thapsigargin, pyrvinium, trifluoperazine, ellipticine, and 0297417-0002B, which in our FDAM were harmful to at least one module, have been reported to show evidence for carcinogenesis/immune suppression activities (Figure 4, Table 1 and Table S3). We view the 41 drugs on the FDAM with no harmful links as candidate therapeutic drugs. The number of modules, or degrees (Table 1), to which a candidate drug was beneficial varied from 1 to 7. There were two degree-7 drugs, phenoxybenzamine and GW-8510, and three degree-5 drugs, thapsigargin, phthalylsulfathiazole, and medrysone (see Table 1 for full details on degrees and drug-module relation). A therapeutic drug compound is a minimum set of drugs culled from the list of candidate therapeutic drugs that covered all the modules. Many compounds could be constructed from the candidate drug list. There were two 2-compnent compounds, phenoxybenzamine+ISP and GW-8510+ISP, and 20 compounds with up to six drug components (Table 2). Barring drug-drug interaction, we predict these compounds to be free of harmful side effects at the intracellular level.

**Table 1:** The list of candidate repurposed drugs.

No	Repurposed drug	D	Module (ES)	Drug function	TTD ID	Carcinogen/immune suppressor	Anticancer agents
1	phenoxybenzamine	7	CC (−0.987), DR (−0.983), At (−0.977), CP (−0.962), Ts (−0.905), ST (−0.886), RM (−0.81)	an α-adrenergic-antagonist	DAP000478	[1, 2]	
2	GW-8510	7	CP (−0.972), ST (−0.936), DR (−0.882), At (−0.867), CC (−0.834), Ts (−0.822), RM (−0.791)	a CDK2 inhibitor that protects hair-loss in chemotheraply	DNC004631		[3]
3	thapsigargin	5	At (−0.918), ST (0.4), Ts (0.521), CP (0.528), RM (0.887)	a nonselective inhibitor of endoplasmic reticulum Ca^2+^ ATPase	DNC014889	[4–7]	[8–11]
4	phthalylsulfathiazole	5	Ts (−0.882), RM (−0.874), CP (−0.767), IS(−0.771), DR (−0.705)	antimicrobial	—		
5	medrysone	5	DR (−0.755), Ts (−0.698), CP (−0.675), IS(−0.686), ST (−0.658)	Adrenocorticotropic hormone drugs	DAP001048		
6	0297417-0002B	4	DR (−0.981), RM (−0.966), Ts (−0.943), IS(0.668)	unknown	—		
7	daunorubicin	4	CC (−0.867), CP (−0.844), RM (−0.8), ST (−0.786)	a chemotherapeutic antibiotic	DAP000788		[12–14]
8	0175029-0000	4	Ts (−0.771), CP (−0.766), ST (−0.698), RM (−0.69)	unknown	—		
9	apigenin	4	DR (−0.896), CP (−0.837), Ts (−0.796), RM (−0.784),	a flavone that have the chemopreventive action in vegetables	DNC004714		[15–18]
10	pyrvinium	3	CC (−0.75), At (−0.694), IS(0.314)	anthelmintic	—		[19, 20]
11	trifluoperazine	3	CC (−0.604), DR (−0.501), IS(0.415)	a typical antipsychotic of the phenothiazine chemical class.	DAP000034	[21]	[22, 23]
12	camptothecin	3	At (−0.953), CP (−0.935), ST (−0.878)	a cytotoxic quinoline alkaloid which inhibits the DNA enzyme topoisomerase I	DNC000385		[24–26]
13	meticrane	3	Ts (−0.726), At (−0.72), RM (−0.713)	Diuretics	—		
14	ellipticine	2	At (−0.827), IS(0.422)	an antineoplastic agent which inhibits the DNA enzyme toposiomerase II	DNC000599	[27–29]	[27–29]
15	8-azaguanine	2	At (−0.87), CP (−0.83)	a purine analog that shows antineoplastic activity	DNC002551		[30, 31]
16	etacrynic acid	2	At (−0.891), CC (−0.875)	GST Inhibitor-2, diuretics	DAP000748		[32]
17	alsterpaullone	2	ST (−0.874), At (−0.866)	CDK inhibitor	DNC000188		[33]
18	vorinostat	2	CP (−0.592), ST (−0.503)	HDAC inhibitor, antineoplastic agent	DAP001082		[34–36]
19	thioguanosine	2	DR (−0.935), Ts (−0.811)	antineoplastic agent	—		[37, 38]
20	sulconazole	2	DR (−0.869), Ts (−0.814)		—		
21	chrysin	2	Ts (−0.934), DR (−0.913)	a naturally occurring flavone, antineoplastic agent	DNC004715		[39, 40]
22	thiostrepton	2	DR (−0.837), Ts (−0.816)	a natural cyclic oligopeptide antibiotic	DNC001438		[41–43]
23	luteolin	2	Ts (−0.856), DR (−0.811)	a flavonoid, antioxidant, anti-inflammatory, and an antineoplastic agent	DNC000896		[44–46]
24	ifenprodil	2	RM (−0.839), Ts (−0.779)	a selective inhibitor of the NMDA receptor, vasodilator	DNC000779		[47]
25	doxazosin	1	At (−0.804)	an α1a-selective alpha blocker, treat high blood pressure	DAP000381		[48–50]
26	cycloserine	1	At (−0.799)	an antibiotic, treatment of tuberculosis	DAP001335		
27	repaglinide	1	At (−0.795)	treatment of type II diabetes	DAP000133		
28	flufenamic acid	1	At (−0.665)	a non-steroidal anti-inflammatory drug.	DNC002446		[51–53]
29	irinotecan	1	At (−0.871)	inhibition of topoisomerase 1, antitumor agent	DAP000647		[54–56]
30	resveratrol	1	CC (−0.627)	a stilbenoid, anticancer, anti-inflammatory	DNC001205		[57–59]
31	withaferin A	1	CC (−0.799)	inhibit agiogenesis and tumorigenesis	—		[60–62]
32	clioquinol	1	CC (−0.719)	an antifungal drug and antiprotozoal drug	DNC011356		[63–65]
33	doxorubicin	1	CC (−0.874)	anthracycline antibiotic, TOP2 inhibitor, antitumor agent	DAP000192		[66–68]
34	bepridil	1	CP (−0.791)	a calcium channel blocker once used to treat angina	DAP000525		[69–71]
35	cloperastine	1	DR (−0.683)	a cough suppressant	—		
36	piperlongumine	1	DR (−0.956)	a natural product which have anti tumor activities	—		[72]
37	morantel	1	IS(−0.839)	Helminthic	—		
38	cetirizine	1	IS(−0.845)	antihistamine, a racemic selective H1 receptor inverse agonist	DAP000323		
39	ginkgolide A	1	IS(−0.834)	Anti-platelet-activating factor	DNC007171		[73]
40	cefalexin	1	IS(−0.744)	cephalosporin antibiotic	DAP000437		
41	triflusal	1	IS(−0.891)	a platelet aggregation inhibitor	—		[74]
42	imipenem	1	IS(−0.791)	an intravenous β-lactam antibiotic	DAP000459		[75, 76]
43	skimmianine	1	RM (−0.815)	Anti-inflammatory, antibacterial	—		
44	6-azathymine	1	ST (−0.813)	Immunodeficiency disease, antitumor agent	—		[77]
45	tyloxapol	1	ST (−0.783)	a nonionic liquid polymer of the alkyl aryl polyether alcohol type	DCL000254		
46	sanguinarine	1	Ts (−0.959)	Anti-bacterial, anti-Trypano-soma and anti-tumor	—		[78–80]

Functional modules (FMs) are those to which a drug has beneficial effects. Drug list is ranked by degree (D), the number of FMs to which a drug is beneficial. Abbreviation for FMs: apoptosis (At), cell cycle (CC), cell proliferation (CP), DNA replication (DR), immune system process (IS), RNA metabolic process (RM), transcription (Ts), signal transduction (ST). TTD: Therapeutic Target Database. References in the last two columns are listed in Table S3.

**Table 2:** Predicted drug compounds for colorectal cancer adenoma.

Code	No. of components	Compound	Ratio of degrees
1	2	phenoxybenzamine + ISP	7∶1
2	2	GW-8510 + ISP	7∶1
3	3*	phthalylsulfathiazole + etacrynic acid + ST	5∶2∶1
4	4	daunorubicin + TDNA + APO + ISP	4∶2∶1∶1
5	4	apigenin + etacrynic acid + ST + ISP	4∶2∶1∶1
6	4	apigenin + alsterpaullone + CC + ISP	4∶2∶1∶1
7	5	camptothecin + ifenprodil + DR + CC + ISP	3∶2∶1∶1∶1
8	5	vorinostat + etacrynic acid + TDNA + RM + ISP	2∶2∶2∶1∶1
9	6	ifenprodil + 8-azaguanine + DR + CC + ST + ISP	2∶2∶1∶1∶1∶1
10	6	ifenprodil + etacrynic acid + DR + CP + CC + ISP	2∶2∶1∶1∶1∶1
11	6	ifenprodil + alsterpaullone + DR + CP + ST + ISP	2∶2∶1∶1∶1∶1
12	6	TDNA + 8-azaguanine + RM + CC + ST + ISP	2∶2∶1∶1∶1∶1
13	6	TDNA + etacrynic acid + RM + CP + ST + ISP	2∶2∶1∶1∶1∶1
14	6	TDNA + alsterpaullone + RM + CC + CP + ISP	2∶2∶1∶1∶1∶1
15	6	TDNA + vorinostat + RM + CP + APO + ISP	2∶2∶1∶1∶1∶1
16	6	vorinostat + etacrynic acid + DR + TR + RM + ISP	2∶2∶1∶1∶1∶1
17	6	vorinostat + ifenprodil + DR + CC + APO + ISP	2∶2∶1∶1∶1∶1
18	6	ifenprodil + 8-azaguanine + DR + CC + ST+ ISP	2∶2∶1∶1∶1∶1
19	6	ifenprodil + alsterpaullone + DR + CC + CP+ ISP	2∶2∶1∶1∶1∶1
20	6	ifenprodil + etacrynic acid + DR + CP + ST+ ISP	2∶2∶1∶1∶1∶1

The eight GO terms (biological function classifications) included are APO, CC, CP, ST, TR, DR, CP, RM and ISP (see abbreviations below). All drugs in the table have ES (enrichment score) <−0.75 (with only one exception). None of the drugs have harmful effects (ES >0) on any of the GO functions. Only compounds with up to 6 components are given. Abbreviations: ISP: immune system process – trifusal or morantel or gingolide or cetirizine or imipenem; APO: apoptosis – irinotecan or doxazosin or cycloserine or repaglinide; CC: cell cycle – doxorubicin or withaferin A; ST: signal transduction – 6-azathymine or tyloxapol; TR: transcription – sanguinarine; DR: DNA replication – piperlongumine; CP: Cell proliferation – bepridil; RM: RNA metabolic process – skimmianine; TDNA: transcription and DNA replication –chrysin or thioguanosine or luteolin or thiostrepton or sulconazole. The “degrees” in “Ratio of degrees” indicate the number of functional modules to which the corresponding component is beneficial.

### Comparison between IGCM and FMCM

#### Stability

As mentioned earlier, the characterization of a drug, namely beneficial or harmful, was much more stable FMCM than in IGCM (Figure 3).

#### Accuracy and specificity

**Figure 5:**
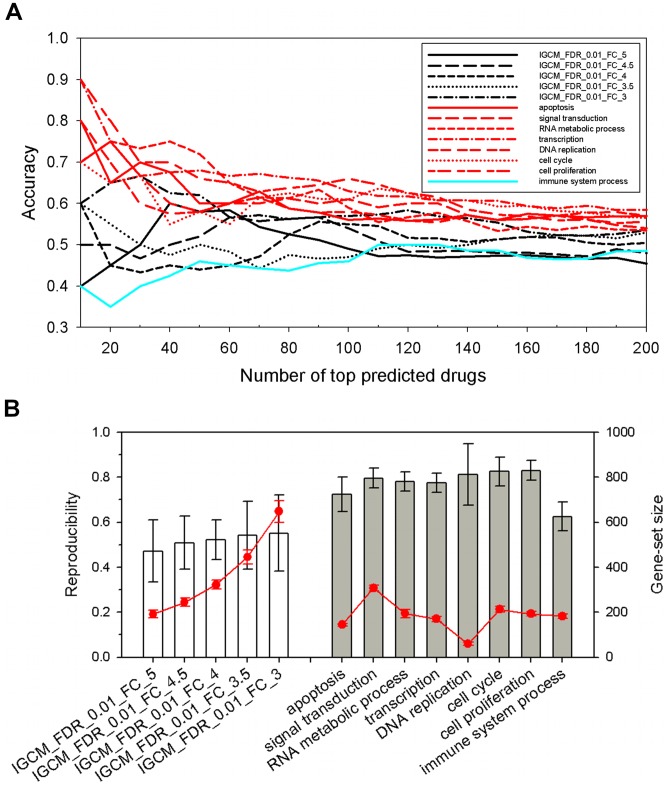
Accuracy and reproducibility in drug prediction. (A) Accuracy is the sum of true positive (predicted beneficial and known anti-tumor agent) and true negative (predicted harmful and known cancer-inducing agent) over sum of predicted beneficial and harmful drugs. IGCM results are in black, and FMCM, in red and cyan. Specificity is given in Figure S5. (B) Reproducibility is the measure of agreement between the selected drugs in two runs using different subsets of microarray data (Materials and Methods). Results shown are averaged over 45 pair-wise comparisons of selected drugs. The five towers on the left are IGCM results for given threshold FC value. The eight towers on the right are FMCM results (FC >2) for the 8 functional modules. Size of querying gene set is given by line in red.

We used anti-tumor agents in the Therapeutic Target Database (TTD) to evaluate the accuracy and specificity (Material and Methods) of the FMCM and IGCM predictions. The “true” drug set in the test was the intersection of TTD and the CMap list, which included about 40% of TTD. For simplicity, we denote by IG3 the IGCM query at FC = 3 and the rest (queries with FC >3) by IGL. We found that FMCM had overall accuracy (Figure 5A) and specificity (Figure S4) similar to IG3 and higher than IGL, except for the immune system process module, where FMCM was worse than IGL.

#### Reproducibility

We tested the reproducibility (Material and Methods) of the drug predictions by repeating 10 times the FMCM and IGCM procedures, each time working on a set of 40 randomly chosen microarrays, 20 each from controls and patients. In the FMCM procedure, GGINs were constructed using DEGs selected by SAM at FDR <0.01 and FC >2, and the selected drug set was the sum of beneficial and harmful drugs. FMCM had significantly higher reproducibility (on average ∼80%) than IGCM (on average ∼50%), except for the module immune system process (on average ∼60%) (Figure 5B, two-sample *t*-test *p*<10^−15^).

#### Clinical application I

**Figure 6:**
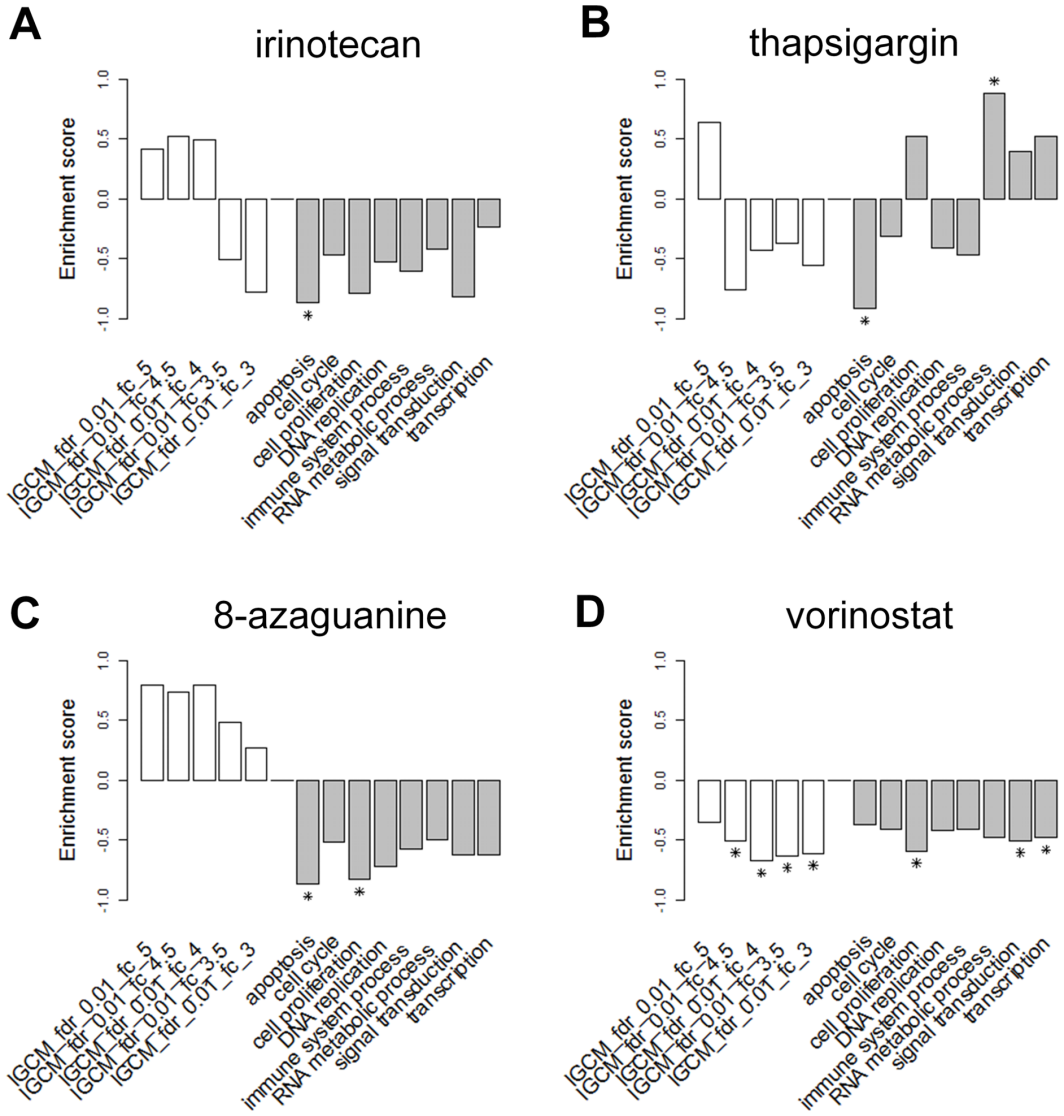
Enrichment scores of known anti-cancer drugs. (A) irinotecan, (B) thapsigargin, (C) 8-azaguanine, and (D) vorinostat. CMap querying gene sets are shown on the horizontal axis. The first five entries from left are whole DEG sets selected by SAM using FDR = 0.01 and FC ranging from 3.0 to 5.0. The rest are the eight functional module selected by GSToP with FC = 2.0. Star indicates permutation *p*-value<0.005.

We took four known anti-cancer drugs, irinotecan (no. 29 in Table 1), thapsigargin (no. 3), 8-azaguanine (no. 15), and vorinostat (no. 18) as examples for comparison (Figure 6). The first drug, irinotecan (trade mark Camptosar), is in current use, in particular in combination with other chemotherapy agents such as 5-fluorouracil and leucovorin in a common colorectal cancer regimen called FOLFIRI [37], was significantly beneficial only to the apoptosis module by FMCM (Figure 6A). Thapsigargin, a known endoplasmic reticulum Ca2+ ATPase inhibitor [38], was both significantly beneficial and harmful to colorectal cancer in FMCM (apoptosis and RNA metabolic process, respectively, Figure 6B), but was neutral IGCM. The third example, 8-azaguanine, a purine analog that exhibits anti-neoplastic activity and have been used in the treatment of acute leukemia [39], was significantly beneficial to the apoptosis and cell proliferation modules in FMCM, but was harmful (not significantly) in all IGCM tests (Figure 6C). Vorinostat (trade mark Zolinza), a member of a larger class of compounds that inhibit histone deacetylases (HDACs), and known to arrest cancer cells epigenetically, was significantly and broadly beneficial in both IGCM (FC from 3 to 4.5) and FMCM (cell proliferation, signal transduction, and transcription) and showed no harmful effects (Figure 6D). These results suggested that FMCM have higher resolution to detect known ant-cancer agents than IGCM.

#### Clinical application II

Using the IGCM and FMCM gene lists we examined the ES versus FC patterns of 27 chemo-drugs, not necessarily specific to colon cancer treatment, listed in the Anatomical Therapeutic Chemical (ATC) classification system (L01 class; anti-neoplastic agents), and partitioned the group by pattern into six types (Figure S5A–F). An overall characterization of our results was that IGL, which represent queries using more stringently selected DEGs, on the one hand and IG3/FMCM on the other tended to give contrast indications to many of the anti-neoplastic agents. In Figure S5A, to which the drug irinotecan belongs, drugs were mostly harmful in the IGL queries but were beneficial in the IG3 and most of the FMCM queries. In Figure S5B, the IGL queries were mostly beneficial and the IG3/FMCM mostly harmful. In Figure S5C and S5D, all IGCM queries (IGL and IG3, with one exception) were beneficial while the FMCM queries had significant harmful components. The most beneficial drug was vorinostat, the single entry in Figure S5E, where all queries were beneficial. The most harmful drugs were the all-harmful celecoxib and paclitaxel, with carmustine and imatinib close behind (Figure S5F). Vorinostat, doxorubicin, daunorubicin, irinotecan – all in Figure S5A – satisfied our stringent criteria for inclusion in Table 1 as components for therapeutic drug compounds.

### Cell viability on single predicted drugs

**Figure 7:**
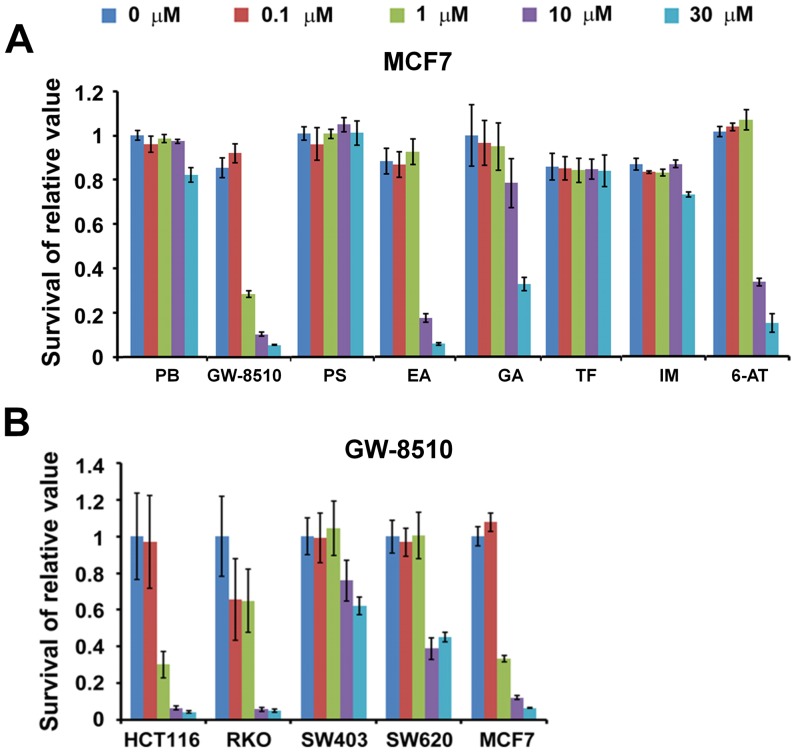
Viability test of colon and breast cancer cells treated with single drug. Tests were conducted on eight drugs: phenoxybenzamine (PB), GW-8510, phthalylsulfathiazole (PS), etacrynic acid (EA), ginkgolide A (GA), triflusal (TF), imipenem (IM), and 6-azathymine (6-AT), with concentrations of 0, 0.1, 1, 10, and 30 µM. (A) Viability of MCF7 on treatment of the eight drugs. (B) Viability of five cell lines on treatment of GW-8510. Colon cancer cells HCT116, RKO, SW403 and SW620, and the breast cancer cell MCF7, were treated with single drug for 5 days. After 5 days, proliferation activities of these cells were detected by Alamar Blue assay.

Eight beneficial drugs from Table 1, phenoxybenzamine (PB; beneficial to 7 functional modules), GW-8510 (GW; 7), phthalylsulfathiazole (PS; 5), etacrynic acid (EA; 2), ginkgolide A (GA; 1), triflusal (TF; 1), imipenem (IM; 1), and 6-azathymine (6-AT; 1), were selected for their commercial availability and degree of beneficence for preliminary cell model experimental validation on five cell lines: colon cancer, HCT116, RKO, SW403, and SW620, and breast cancer, MCF7. GW had the strongest effect on the cell lines and MCF7 was the cell line most susceptible to the tested drugs (Figure 7). GW, EA, GA, and 6-AT could selectively or broadly inhibit cell viability on the cell lines. GW, a known CDK2 inhibitor used in protection of hair-loss in chemotherapy, exhibited strong inhibitions against HCT116 and MCF7, moderate effects against RKO and SW620, and weak effects against SW602. EA, GA, and 6-AT moderately to weakly inhibited the viability of MCF7 (Table 3).

**Table 3:** Inhibitory effects of single predicted drugs on colon cancer and breast cancer cell lines.

	Half maximal inhibitory concentration (IC50) (µM)				
Drugs	HCT116	RKO	SW403	SW620	MCF7
phenoxybenzamine	–	–	–	–	–
GW-8510	0.7	3.3	>30	8.4	0.8
phthalylsulfathiazole	–	–	–	–	–
etacrynic acid	–	–	–	–	6.80
ginkgolide A	–	–	–	–	22.5
triflusal	–	–	–	–	–
imipenem	–	–	–	–	–
6-azathymine	–	–	–	–	7.9

–: not detected from 0.1 to 30 µM.

### Microarray results and test of the perturbagen concept

**Figure 8:**
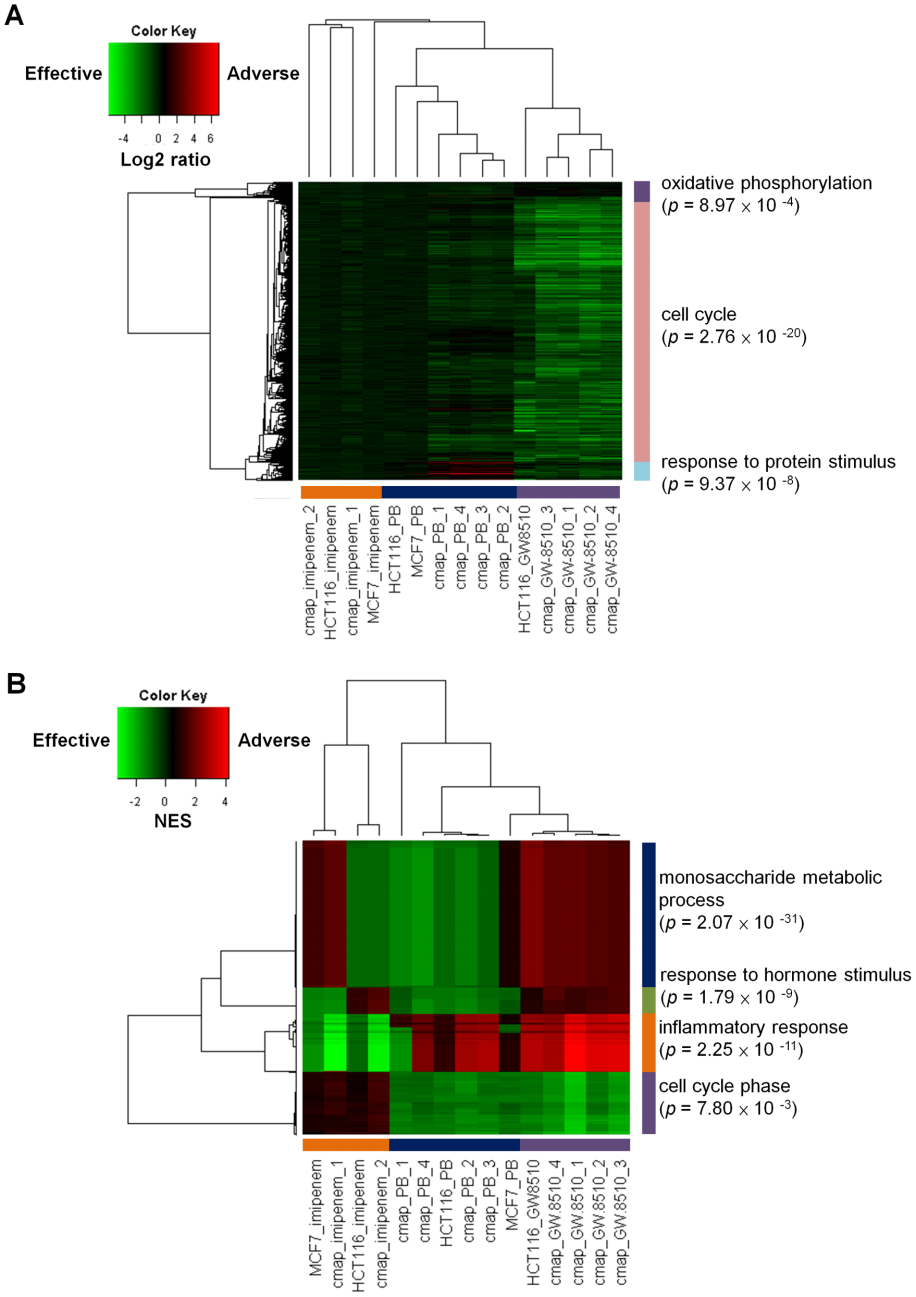
Clustering of genomic profiles of drug-treated cancer cell lines HCT116 and MCF7. (A) Individual gene approach (IGA). (B) Gene-set approach (GSA). Cell lines were treated with three drugs: GW-8510, phenoxybenzamine (PB), and imipenem. Entries marked “cmap” were microarray drug treatment genomic profiles of MCF7 taken from the CMap. Others were from drug treatment microarray experiments (Affymetrix U219 (PrimeView) platform) conducted for the present study, where the same experimental protocol used in CMap were followed: averaged over three dosages of 10 M, 11.8 M, 13.4 M; treatment time 6 hours after overnight culture. Heatmaps were results of two-way hierarchical clustering.

We tested the implicit perturbagen assumption [17] that CMap data on gene expression profiles from drug treatments on one cell line (MCF7) are useful for drawing inferences more generally on the effects of the drug, in particular, its effect on different cell lines. We generated five microarray global gene expression profiles, of PB and IM on HCT116 and MCF7, and of GW on HCT116 (the efficacy of GW on MCF7 is similar). As control we extracted from the CMap database 10 corresponding datasets of the three drugs on MCF7 (4, 4, and 2 datasets from PB, GW, and IM, respectively). We carried out separate two-way hierarchical clustering of the 15 profiles employing the IGA and GSA procedures. In spite of different cell lines, microarray platforms, and laboratory conditions, the samples, especially the five GW administered samples, clustered more according to drugs than not in both procedures (Figure 8). The outstanding qualitative difference between the IGA and GSA procedures was that the IGA heatmap was dominated by a single GO term, cell cycle (Figure 8A, Table S4, S5, S6), whereas the GSA heatmap was characterized by four terms: monosaccharide metabolic process, response to hormone stimulus, inflammatory response, and cell cycle phases (Figure 8B, Table S7, S8, S9, S10). The IGA heatmap corroborates the result mentioned earlier, that GW, alone among all the drugs, had high negative impact on cell survival. The GSA heatmap allowed the effects of smaller gene sets to be displayed and had a closer correspondence to our FMCM approach to drug selection. For example, it provided an independent support that IM, but not the other drugs tested, exhibited a strong beneficial effect on the immune system process in colorectal cancer cells (Table 1).

## Discussion

CMap has been widely applied to drug discovery for treatment of complex diseases, including cancer. Methodologies employed for applying CMap for this purpose are simply variations of a [17]. The important differences between the FMCM procedure used here and IGCM include: (i) In addition to differential expression, genes selected from querying CMap were screened by the GSToP procedure making use of GGINs [26]; (ii) functional modules (FMs) were built from selected genes and used for separate querying of CMap to select function specific beneficial drugs (Table 1); (iii) querying results were used to form drug compounds, each compound being a minimum set of drugs that collectively were beneficial to all the FMs and not harmful to any FM (Table 2).

Our tests showed FMCM to perform better than IGCM in terms of prediction stability (Figure 3), accuracy (Figure 5A), specificity (Figure S4), and reproducibility (Figure 5B). In short, FMCM was much more robust than IGCM in drugs selected. One reason for the relative robustness of FMCM prediction over IGCM may be that most modern drugs were designed to affect a specific biological function, say, by targeting a transcription factor, not to affect all functions. An FM-drug association is therefore expected to be more stable than that between the whole DEG set and the drug.

Because IGCM does not test drugs for function specific harmfulness, drugs selected by IGCM may be deemed overall beneficial yet still harmful to some functions, or vice versa. This was the case for three well-known drugs used in the cancer chemotherapy listed in the CMap database: thapsigargin, 8-azaguanine, and irinotecan (Figure 6).

Drugs selected by FMCM had high sensitivity and low false-positive rate. Of the total 46 candidate drugs (Table 1), 41 were entirely beneficial and five were harmful to some FMs. Thirty of 46 drugs have been reported in the literature to have properties related to cancer. Of the 41 putative entirely beneficial drugs, 25 have been reported to have anticancer properties and one has been reported to be carcinogenic (or mutagenic). Of the five putative partly harmful drugs, four have been reported to be both anticancer and carcinogenic.

Phenoxybenzamine, an α-adrenergic-antagonist, was our only false-positive case. We identified it as a degree-7 beneficial drug, but there has been no literature suggesting its anti-tumorigenicity. Instead, there are two clinical studies suggesting possible carcinogenic effects in patients [40], [41].

A novel feature of the FMCM approach was its ability to discover intracellular harmful side effect in agents known to be anti-cancer. This is not surprising given the widespread practice of targeting specific biomolecule in drug designs when it is now known that the typical regulatory relation between transcription factors and biological networks is many-to-many. We identified pyrvinium, trifluoperazine, ellipticine, and 0297417-0002B to be harmful to immune system process but otherwise beneficial. The first three – there is no literature on 0297417-0002B – has been reported to have anti-tumor properties but also have lethal effects in cell or animal models (Table 1). We identified thapsigargin to be beneficial to the apoptosis FM but broadly harmful to the signal transduction, transcription, cell proliferation, and RNA metabolic process FMs. This drug has been reported to have the ability to promote apoptosis on prostate and breast cancer cells but also to stimulate cell growth in mouse keratinocyte models (Table 1) [42]–[47].

Based on our *in silico* screening and cell viability experiments, we selected the four drugs, GW-8510 (GW, no. 2 in Table 1), etacrynic acid (EA, no. 16), ginkgolide A (GA, no. 39), and 6-azathymine (6-AT, no. 44), as potential therapeutic agents for colorectal adenoma. GW, which exhibited clear inhibitory effects against colon cell growth (Figure 7) in our viability experiments, is a cyclin-dependent kinase 2 (CDK2) inhibitor used for preventing hair loss in chemotherapy. It was suggested that the observed antitumor efficacy of GW's derives from its inhibition of tumor growth via cell cycle control [48], a suggestion supported by our *in silico* study. EA is a potent inhibitor of glutathione *S*-transferase (GST) family members and has been used to treat high blood pressure and swelling caused by kidney failure. It has been suggested that EA may inhibit cell growth and induce cancer cell death through apoptosis [49]–[51], a notion that correlated well with our findings (Figure 4). In our analysis the ES's for EA were −0.891 and −0.875 versus the apoptosis and cell cycle modules, respectively (Table 1). EA has also been reported to have therapeutic potential in cancer therapy by reversing drug resistance [52]–[54]. GA, a *Ginkgo biloba* leaf extract, has been used for treatment in a wide variety of cognitive and vascular disorders, including dementia and peripheral arterial occlusive diseases [55]–[57]. Its structural homolog Ginkgolide B has been reported to possess anti-inflammatory anti-allergic, anti-oxidant, anti-cancer, and neuroprotective effects [58]. In our analysis GA had ES = −0.834 against the immune system process module (Table 1). Recent studies conducted with various molecular, cellular, and animal model experiments have concluded that Ginkgolide B may have chemopreventive abilities associated with anti-angiogenic, antioxidant, and gene-regulatory events [59]–[62]. 6-AT, an analog of thymine, has been shown to inhibit the pathway of biosynthesis of nuclear acids in cancer tissues [63], [64]. It had ES = −0.813 against the signal transduction module (Table 1) and inhibited tumor growth in the cell line MCF7 (Figure 7). Our study showed that the pair GW and GA combined would yield beneficial effects on all eight FMs (Table 2).

**Figure 9:**
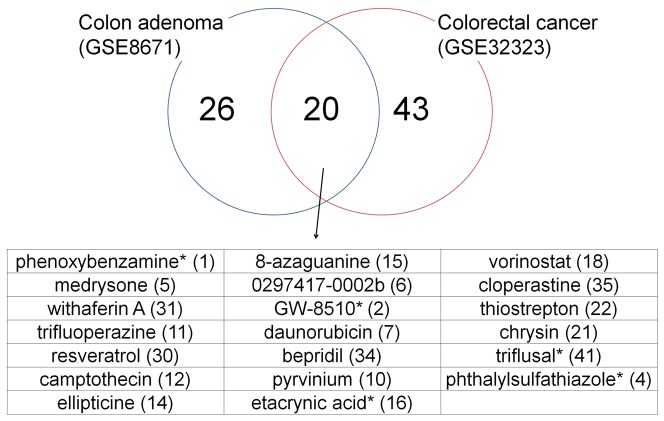
Overlap of candidate repurposed drug sets curated from colon adenoma and colorectal cancer data sets. Numbers in brackets correspond to those given in the first column of Table 1, which lists the drug set for colon adenoma. The overlap includes 9 of the 13 drugs in Table 1 with degrees not less than 3, and 5 of the 8 drugs selected for cell viability tests marked by “*”.

We were not able to find another gene expression microarray set on colon adenoma that passed our quality test (Principal Component Analysis, Figure S1). We did find a dataset on colorectal cancer (CRC) that did, from GEO database (accession number: GSE32323). The dataset, Affymetrix HG-U133 plus 2.0 arrays, contained 17 pairs of cancer and healthy colon tissues from CRC patients [65]. We subjected the set to the same IGCM and FMCM analyses as we did the adenoma data. For the predicted repurposed candidate drugs, FMCM performed better than IGCM in stability and reproducibility, and was comparable with IGCM in accuracy and specificity. Through FMCM we selected 43 candidate drugs for CRC. Among this drug set, 20 also belonged to the set of 46 drugs for adenoma (Figure 9). This overlap set of 20 drugs included 9 of the 13 adenoma drugs with degrees equal to or greater than 3 (Table 1). Given that IGCM-based drug screening for disease specific but different data sets is known to lead to drug sets that are highly variable, and that colon adenoma and CRC are related but not the same conditions, the degrees of overlap between the colon adenoma and CRC drug sets are encouraging. The overlap set contained 5 of the 8 adenoma drugs selected for cell viability tests. This is significant because the tests were conducted prior to the analysis of CRC data.

There are limitations inherent to our approach. It depends on the availability of gene expression profiles of drugs; many FDA-approved drugs are not among the more than 1,000 compounds with profiles given in CMap database 2.0 release, the version used in this work; the implicit perturbagen assumption that the drug-specific genomic profiles given by CMap are only mildly dependent on the specific (mostly breast cancer) cell lines requires validation in every application; the reliability of our results depends on the quality and accuracies of patient gene expression profiles, PPI, and GO data; and, specific to this work, how the drug screening results may depend on the selection of FMs for drug screening has not been thoroughly studied.

Our results need further experimental validation because therapeutic efficacy of a drug is always more complex than just a simple matching of expression profiles, even when conducted in the refined fashion of FMCM. In addition, not known at this stage are how components in a compound (Table 2) would interact with each other and how the interaction would impact its predicted property. Such effects can only be assessed in animal model tests when the selected drugs are applied in compound form.

These limitations are common to all CMap-based or similar drug discovery methods, yet our method has merits not possessed by other screening methods. We believe our method is useful for drug discovery for therapeutic systems treatment not only for colorectal adenoma, but also for other types of cancer as well as for other complex diseases and conditions.

## Supporting Information

Figure S1 **Assessment of chip quality and sample classification of colorectal adenocarcinoma paired patients.** (A) Principle Component Analysis. The first component, with about 35% variances in data, was sufficient to correctly partition the samples, 32 of adenoma patients and their 32 parental normal tissues, into two groups. (B) Two-way hierarchical clustering analysis. On the side, 2,164 differentially expressed genes (DEGs) were used for clustering. The DEGs, including 964 up-regulated and 1,200 down-regulated genes, were selected by the SAM algorithm with thresholds for false discovery rate (FDR) <0.05 and fold change (FC) >2. On the top, green bar covers the normal tissue and blue bar, adenocarcinoma.
(TIF)

Figure S2 **The gene-gene networks constructed using gene expression data from normal and colorectal adenoma patients.** There are 1,684 genes and 2,017 links in the normal network, and 1,792 genes and 2,656 links in the adenoma network. Genes assigned to over-represented biological Gene Ontology terms are highlighted in term specific color.
(TIF)

Figure S3 **Drugs selected by standard application of CMap, or IGCM, using different fold change (FC) thresholds.** Sum of up- and down-regulated genes given under each FC threshold constituted the querying gene set. Drugs listed are those predicted to be beneficial. Red arrow indicates known TTD anti-cancer agents that coincidentally all changed from beneficial at FC = 3 to harmful at FC = 3.5. Vorinostat was the only drug selected at FC >3, 3.5, 4.0, and 4.5; it was also selected in the FMCM procedure.
(TIF)

Figure S4 **Specificity of predicted drugs.** Specificity is true negative (known cancer-inducing agent predicted to be harmful) over all drugs predicted to be harmful; higher specificity implies lower false positive. Seven of the eight FMCM results (red), except immune systems process (cyan), have higher specificities than the five IGCM results (black).
(TIF)

Figure S5 **Enrichment scores of 27 chemo-drugs.** The 27 chemo-drugs, selected from the L01 class (antineoplastic agents) in the Anatomical Therapeutic Chemical system, are not specific to colon cancer treatment. The ES is those from five IGCM (FC threshold 3 to 5) and eight FMCM runs (FC >0.2). Solid symbol indicates an ES with permutation *p* value<0.05. The 27 drugs are clustered into six groups according to overall pattern.
(TIF)

Table S1 **Gene ontology enrichment analysis for functional modules.**
(XLS)

Table S2 **Gene signature tags used in the FMCM program.**
(XLS)

Table S3 **References listed in** **Table 1** **.**
(XLS)

Table S4 **GO terms analysis for genes in the lightblue block in the IGA heatmap (** **Figure 8A** **).** Top-10 gene ontology annotation clusters were determined by DAVID [36].
(XLS)

Table S5 **GO terms analysis for genes in the pink block in the IGA heatmap (** **Figure 8A** **).** Top-10 gene ontology annotation clusters were determined by DAVID [36].
(XLS)

Table S6 **GO terms analysis for genes in the purple block in the IGA heatmap (** **Figure 8A** **).** Top-10 gene ontology annotation clusters were determined by DAVID [36].
(XLS)

Table S7 **GO terms analysis for genes in the green block in the GSA heatmap (** **Figure 8B** **).** Top-10 gene ontology annotation clusters were determined by DAVID [36].
(XLS)

Table S8 **GO terms analysis for genes in the blue block in the GSA heatmap (** **Figure 8B** **).** Top-10 gene ontology annotation clusters were determined by DAVID [36].
(XLS)

Table S9 **GO terms analysis for genes in the orange block in the GSA heatmap (** **Figure 8B** **).** Top-10 gene ontology annotation clusters were determined by DAVID [36].
(XLS)

Table S10 **GO terms analysis for genes in the purple block in the GSA heatmap (** **Figure 8B** **).** Top-10 gene ontology annotation clusters were determined by DAVID [36].
(XLS)

## References

[1] Sleigh SH, Barton CL (2010) Repurposing Strategies for Therapeutics. Pharmaceutical Medicine 24: 151–159 110.2165/11536770-000000000-000000000.

[2] Kamb A, Wee S, Lengauer C (2007) Why is cancer drug discovery so difficult? Nat Rev Drug Discov 6: 115–120. 17159925 10.1038/nrd2155

[3] Chong CR, Sullivan DJ Jr (2007) New uses for old drugs. Nature 448: 645–646. 17687303 10.1038/448645a

[4] Issa AM, Phillips KA, Van Bebber S, Nidamarthy HG, Lasser KE, et al (2007) Drug withdrawals in the United States: a systematic review of the evidence and analysis of trends. Curr Drug Saf 2: 177–185. 18690965 10.2174/157488607781668855

[5] Hill KD, Wee R (2012) Psychotropic Drug-Induced Falls in Older People A Review of Interventions Aimed at Reducing the Problem. Drugs & Aging 29: 15–30. 22191720 10.2165/11598420-000000000-00000

[6] Goldkind L, Laine L (2006) A systematic review of NSAIDs withdrawn from the market due to hepatotoxicity: lessons learned from the bromfenac experience. Pharmacoepidemiol Drug Saf 15: 213–220. 16456879 10.1002/pds.1207

[7] Imming P, Sinning C, Meyer A (2006) Opinion - Drugs, their targets and the nature and number of drug targets. Nature Reviews Drug Discovery 5: 821–834. 17016423 10.1038/nrd2132

[8] Merino A, Bronowska AK, Jackson DB, Cahill DJ (2010) Drug profiling: knowing where it hits. Drug Discov Today 15: 749–756. 20601095 10.1016/j.drudis.2010.06.006

[9] Rettig RA (2006) The war on cancer: An anatomy of failure, a blueprint for the future. Health Affairs 25: 1446–1447.

[10] Kreeger PK, Lauffenburger DA (2010) Cancer systems biology: a network modeling perspective. Carcinogenesis 31: 2–8. 19861649 10.1093/carcin/bgp261 PMC2802670

[11] Hanahan D, Weinberg RA (2011) Hallmarks of cancer: the next generation. Cell 144: 646–674. 21376230 10.1016/j.cell.2011.02.013

[12] Schadt EE, Friend SH, Shaywitz DA (2009) A network view of disease and compound screening. Nat Rev Drug Discov 8: 286–295. 19337271 10.1038/nrd2826

[13] Berger SI, Iyengar R (2009) Network analyses in systems pharmacology. Bioinformatics 25: 2466–2472. 19648136 10.1093/bioinformatics/btp465 PMC2752618

[14] Hopkins AL (2008) Network pharmacology: the next paradigm in drug discovery. Nat Chem Biol 4: 682–690. 18936753 10.1038/nchembio.118

[15] Hornberg JJ, Bruggeman FJ, Westerhoff HV, Lankelma J (2006) Cancer: a Systems Biology disease. Biosystems 83: 81–90. 16426740 10.1016/j.biosystems.2005.05.014

[16] Oprea TI, Mestres J (2012) Drug repurposing: far beyond new targets for old drugs. AAPS J 14: 759–763. 22826034 10.1208/s12248-012-9390-1 PMC3475856

[17] Lamb J, Crawford ED, Peck D, Modell JW, Blat IC, et al (2006) The Connectivity Map: using gene-expression signatures to connect small molecules, genes, and disease. Science 313: 1929–1935. 17008526 10.1126/science.1132939

[18] Garman KS, Acharya CR, Edelman E, Grade M, Gaedcke J, et al (2008) A genomic approach to colon cancer risk stratification yields biologic insights into therapeutic opportunities. Proc Natl Acad Sci U S A 105: 19432–19437. 19050079 10.1073/pnas.0806674105 PMC2592987

[19] Huang L, Zhao S, Frasor JM, Dai Y (2011) An integrated bioinformatics approach identifies elevated cyclin E2 expression and E2F activity as distinct features of tamoxifen resistant breast tumors. PLoS One 6: e22274. 21789246 10.1371/journal.pone.0022274 PMC3137633

[20] Wang G, Ye Y, Yang X, Liao H, Zhao C, et al (2011) Expression-based in silico screening of candidate therapeutic compounds for lung adenocarcinoma. PLoS One 6: e14573. 21283735 10.1371/journal.pone.0014573 PMC3024967

[21] Dudley JT, Sirota M, Shenoy M, Pai RK, Roedder S, et al (2011) Computational repositioning of the anticonvulsant topiramate for inflammatory bowel disease. Sci Transl Med 3: 96ra76. 10.1126/scitranslmed.3002648 PMC3479650 21849664

[22] Sirota M, Dudley JT, Kim J, Chiang AP, Morgan AA, et al (2011) Discovery and preclinical validation of drug indications using compendia of public gene expression data. Sci Transl Med 3: 96ra77. 10.1126/scitranslmed.3001318 PMC3502016 21849665

[23] Shigemizu D, Hu ZJ, Hung JH, Huang CL, Wang YJ, et al (2012) Using Functional Signatures to Identify Repositioned Drugs for Breast, Myelogenous Leukemia and Prostate Cancer. Plos Computational Biology 8. 10.1371/journal.pcbi.1002347 PMC3276504 22346740

[24] Schoeberl B, Pace EA, Fitzgerald JB, Harms BD, Xu L, et al (2009) Therapeutically targeting ErbB3: a key node in ligand-induced activation of the ErbB receptor-PI3K axis. Sci Signal 2: ra31. 19567914 10.1126/scisignal.2000352

[25] Kumar N, Afeyan R, Kim HD, Lauffenburger DA (2008) Multipathway model enables prediction of kinase inhibitor cross-talk effects on migration of Her2-overexpressing mammary epithelial cells. Mol Pharmacol 73: 1668–1678. 18349105 10.1124/mol.107.043794 PMC4329972

[26] Chung FH, Lee HH, Lee HC (2013) ToP: A Trend-of-Disease-Progression Procedure Works Well for Identifying Cancer Genes from Multi-State Cohort Gene Expression Data for Human Colorectal Cancer. PLoS One 8: e65683. 23799036 10.1371/journal.pone.0065683 PMC3683052

[27] Sabates-Bellver J, Van der Flier LG, de Palo M, Cattaneo E, Maake C, et al (2007) Transcriptome profile of human colorectal adenomas. Mol Cancer Res 5: 1263–1275. 18171984 10.1158/1541-7786.MCR-07-0267

[28] Keshava Prasad TS, Goel R, Kandasamy K, Keerthikumar S, Kumar S, et al (2009) Human Protein Reference Database–2009 update. Nucleic Acids Res 37: D767–772. 18988627 10.1093/nar/gkn892 PMC2686490

[29] Ashburner M, Ball CA, Blake JA, Botstein D, Butler H, et al (2000) Gene ontology: tool for the unification of biology. The Gene Ontology Consortium. Nature genetics 25: 25–29. 10802651 10.1038/75556 PMC3037419

[30] Zhu F, Shi Z, Qin C, Tao L, Liu X, et al (2012) Therapeutic target database update 2012: a resource for facilitating target-oriented drug discovery. Nucleic Acids Res 40: D1128–1136. 21948793 10.1093/nar/gkr797 PMC3245130

[31] Subramanian A, Tamayo P, Mootha VK, Mukherjee S, Ebert BL, et al (2005) Gene set enrichment analysis: a knowledge-based approach for interpreting genome-wide expression profiles. Proc Natl Acad Sci U S A 102: 15545–15550. 16199517 10.1073/pnas.0506580102 PMC1239896

[32] Tusher VG, Tibshirani R, Chu G (2001) Significance analysis of microarrays applied to the ionizing radiation response. Proc Natl Acad Sci U S A 98: 5116–5121. 11309499 10.1073/pnas.091062498 PMC33173

[33] Alexa A, Rahnenfuhrer J, Lengauer T (2006) Improved scoring of functional groups from gene expression data by decorrelating GO graph structure. Bioinformatics 22: 1600–1607. 16606683 10.1093/bioinformatics/btl140

[34] Irizarry RA, Hobbs B, Collin F, Beazer-Barclay YD, Antonellis KJ, et al (2003) Exploration, normalization, and summaries of high density oligonucleotide array probe level data. Biostatistics 4: 249–264. 12925520 10.1093/biostatistics/4.2.249

[35] Smyth GK (2004) Linear models and empirical bayes methods for assessing differential expression in microarray experiments. Stat Appl Genet Mol Biol 3: Article3. 16646809 10.2202/1544-6115.1027

[36] Huang da W, Sherman BT, Lempicki RA (2009) Systematic and integrative analysis of large gene lists using DAVID bioinformatics resources. Nat Protoc 4: 44–57. 19131956 10.1038/nprot.2008.211

[37] Douillard JY, Cunningham D, Roth AD, Navarro M, James RD, et al (2000) Irinotecan combined with fluorouracil compared with fluorouracil alone as first-line treatment for metastatic colorectal cancer: a multicentre randomised trial. Lancet 355: 1041–1047. 10744089 10.1016/s0140-6736(00)02034-1

[38] Rogers TB, Inesi G, Wade R, Lederer WJ (1995) Use of thapsigargin to study Ca2+ homeostasis in cardiac cells. Bioscience Reports 15: 341–349. 8825036 10.1007/BF01788366

[39] Frei E 3rd, Holland JF, Schneiderman MA, Pinkel D, Selkirk G, et al (1958) A comparative study of two regimens of combination chemotherapy in acute leukemia. Blood 13: 1126–1148. 13596417

[40] Vaidyanathan S, Mansour P, Soni BM, Hughes PL, Singh G (2006) Chronic lymphocytic leukaemia, synchronous small cell carcinoma and squamous neoplasia of the urinary bladder in a paraplegic man following long-term phenoxybenzamine therapy. Spinal Cord 44: 188–191. 16130025 10.1038/sj.sc.3101789

[41] Brambilla G, Martelli A (2006) Genotoxicity and carcinogenicity studies of antihypertensive agents. Mutat Res 612: 115–149. 16458045 10.1016/j.mrrev.2005.12.002

[42] Pereira M, Millot JM, Sebille S, Manfait M (2002) Inhibitory effects of extracellular Mg2+ on intracellular Ca2+ dynamic changes and thapsigargin-induced apoptosis in human cancer MCF7 cells. Mol Cell Biochem 229: 163–171. 11936842 10.1023/a:1017972622312

[43] Tombal B, Weeraratna AT, Denmeade SR, Isaacs JT (2000) Thapsigargin induces a calmodulin/calcineurin-dependent apoptotic cascade responsible for the death of prostatic cancer cells. Prostate 43: 303–317. 10861750 10.1002/1097-0045(20000601)43:4<303::aid-pros10>3.0.co;2-v

[44] Lin XS, Denmeade SR, Cisek L, Isaacs JT (1997) Mechanism and role of growth arrest in programmed (apoptotic) death of prostatic cancer cells induced by thapsigargin. Prostate 33: 201–207. 9365549 10.1002/(sici)1097-0045(19971101)33:3<201::aid-pros9>3.0.co;2-l

[45] Hakii H, Fujiki H, Suganuma M, Nakayasu M, Tahira T, et al (1986) Thapsigargin, a histamine secretagogue, is a non-12-O-tetradecanoylphorbol-13-acetate (TPA) type tumor promoter in two-stage mouse skin carcinogenesis. J Cancer Res Clin Oncol 111: 177–181. 2426275 10.1007/BF00389230 PMC12253923

[46] Harmon CS, Ducote J, Xiong Y (1996) Thapsigargin induces rapid, transient growth inhibition and c-fos expression followed by sustained growth stimulation in mouse keratinocyte cultures. J Invest Dermatol 107: 188–194. 8757761 10.1111/1523-1747.ep12329592

[47] Lee DI, Sumbilla C, Lee M, Natesavelalar C, Klein MG, et al (2007) Mechanisms of resistance and adaptation to thapsigargin in androgen-independent prostate cancer PC3 and DU145 cells. Arch Biochem Biophys 464: 19–27. 17475205 10.1016/j.abb.2007.03.040

[48] Kent LL, Hull-Campbell NE, Lau T, Wu JC, Thompson SA, et al (1999) Characterization of novel inhibitors of cyclin-dependent kinases. Biochem Biophys Res Commun 260: 768–774. 10403840 10.1006/bbrc.1999.0891

[49] Aizawa S, Ookawa K, Kudo T, Asano J, Hayakari M, et al (2003) Characterization of cell death induced by ethacrynic acid in a human colon cancer cell line DLD-1 and suppression by N-acetyl-L-cysteine. Cancer Sci 94: 886–893. 14556662 10.1111/j.1349-7006.2003.tb01371.x PMC11160201

[50] Seyfried J, Soldner F, Schulz JB, Klockgether T, Kovar KA, et al (1999) Differential effects of L-buthionine sulfoximine and ethacrynic acid on glutathione levels and mitochondrial function in PC12 cells. Neurosci Lett 264: 1–4. 10319999 10.1016/s0304-3940(99)00107-x

[51] Lu D, Liu JX, Endo T, Zhou H, Yao S, et al (2009) Ethacrynic acid exhibits selective toxicity to chronic lymphocytic leukemia cells by inhibition of the Wnt/beta-catenin pathway. PLoS One 4: e8294. 20011538 10.1371/journal.pone.0008294 PMC2789382

[52] Rhodes T, Twentyman PR (1992) A study of ethacrynic acid as a potential modifier of melphalan and cisplatin sensitivity in human lung cancer parental and drug-resistant cell lines. Br J Cancer 65: 684–690. 1316774 10.1038/bjc.1992.145 PMC1977394

[53] Lacreta FP, Brennan JM, Nash SL, Comis RL, Tew KD, et al (1994) Pharmakokinetics and bioavailability study of ethacrynic acid as a modulator of drug resistance in patients with cancer. J Pharmacol Exp Ther 270: 1186–1191. 7932170

[54] Tew KD, Bomber AM, Hoffman SJ (1988) Ethacrynic acid and piriprost as enhancers of cytotoxicity in drug resistant and sensitive cell lines. Cancer Res 48: 3622–3625. 3288331

[55] Woelkart K, Feizlmayr E, Dittrich P, Beubler E, Pinl F, et al (2010) Pharmacokinetics of bilobalide, ginkgolide A and B after administration of three different Ginkgo biloba L. preparations in humans. Phytother Res 24: 445–450. 20041430 10.1002/ptr.3074

[56] Ahlemeyer B, Krieglstein J (2003) Pharmacological studies supporting the therapeutic use of Ginkgo biloba extract for Alzheimer's disease. Pharmacopsychiatry 36 (Suppl 1) S8–14. 13130383 10.1055/s-2003-40454

[57] Shi C, Liu J, Wu F, Yew DT (2010) Ginkgo biloba extract in Alzheimer's disease: From action mechanisms to medical practice. Int J Mol Sci 11: 107–123. 20162004 10.3390/ijms11010107 PMC2820992

[58] Xia SH, Fang DC (2007) Pharmacological action and mechanisms of ginkgolide B. Chin Med J (Engl) 120: 922–928. 17543184

[59] Aponte MM, Kwong J, Mok SC, Berkowitz RS, Cramer DW, et al. (2006) Gingkolide may prevent ovarian cancer through the platelet activating factor receptor (PAFR) pathway. AACR Meeting Abstracts 2006: : A106–.

[60] Jiang W, Qiu W, Wang Y, Cong Q, Edwards D, et al (2011) Ginkgo may prevent genetic-associated ovarian cancer risk: multiple biomarkers and anticancer pathways induced by ginkgolide B in BRCA1-mutant ovarian epithelial cells. Eur J Cancer Prev 20: 508–517. 21857521 10.1097/CEJ.0b013e328348fbb7

[61] Jiang W, Cong Q, Wang Y, Ye B, Xu C (2012) Ginkgo May Sensitize Ovarian Cancer Cells to Cisplatin: Antiproliferative and Apoptosis-Inducing Effects of Ginkgolide B on Ovarian Cancer Cells. Integr Cancer Ther 10.1177/1534735411433833 22505596

[62] DeFeudis FV, Papadopoulos V, Drieu K (2003) Ginkgo biloba extracts and cancer: a research area in its infancy. Fundam Clin Pharmacol 17: 405–417. 12914542 10.1046/j.1472-8206.2003.00156.x

[63] Prusoff WH, Holmes WL, Welch AD (1954) Biological investigations of 6-azathymine, a thymine analog. Cancer Res 14: 570–574. 13199799

[64] Ellison RR, Tan CT, Murphy ML, Krakoff IH (1960) [Clinical investigations of 6-azathymine: a thymine analog]. Cancer Res 20: 435–442. 13848285

[65] Khamas A, Ishikawa T, Shimokawa K, Mogushi K, Iida S, et al (2012) Screening for epigenetically masked genes in colorectal cancer Using 5-Aza-2′-deoxycytidine, microarray and gene expression profile. Cancer Genomics Proteomics 9: 67–75. 22399497

