# Supplementary figures and images for: Functional Module Connectivity Map (FMCM): A Framework for Searching Repurposed Drug Compounds for Systems Treatment of Cancer and an Application to Colorectal Adenocarcinoma
Source: PLoS One. 2014 Jan 27;9(1):e86299. doi: 10.1371/journal.pone.0086299 (PMC3903539; doi:10.1371/journal.pone.0086299)

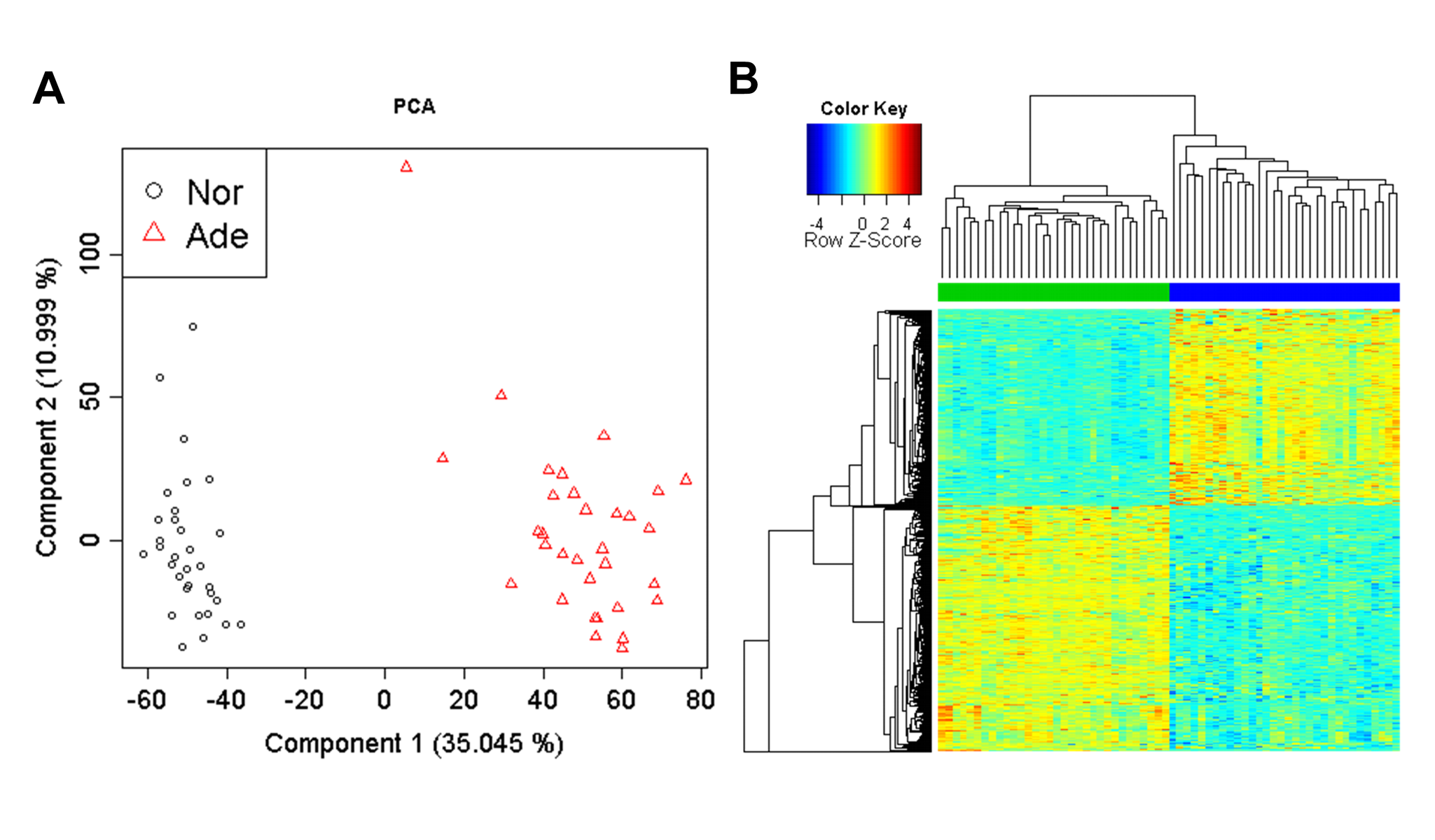

Supplement: Figure S1 — Assessment of chip quality and sample classification of colorectal adenocarcinoma paired patients. (A) Principle Component Analysis. The first component, with about 35% variances in data, was sufficient to correctly partition the samples, 32 of adenoma patients and their 32 parental normal tissues, into two groups. (B) Two-way hierarchical clustering analysis. On the side, 2,164 differentially expressed genes (DEGs) were used for clustering. The DEGs, including 964 up-regulated and 1,200 down-regulated genes, were selected by the SAM algorithm with thresholds for false discovery rate (FDR) <0.05 and fold change (FC) >2. On the top, green bar covers the normal tissue and blue bar, adenocarcinoma. (TIF) [file pone.0086299.s001.tif]

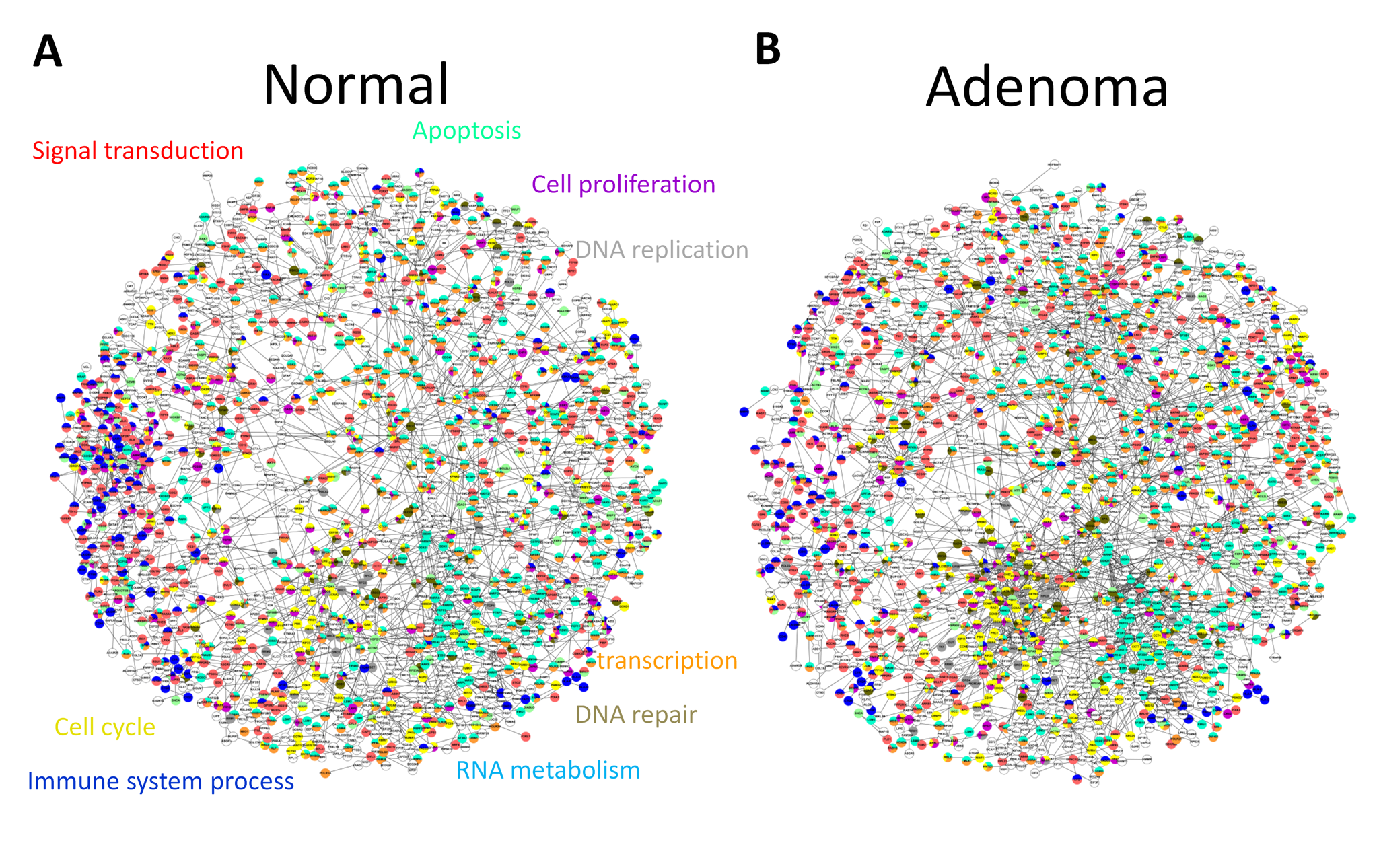

Supplement: Figure S2 — The gene-gene networks constructed using gene expression data from normal and colorectal adenoma patients. There are 1,684 genes and 2,017 links in the normal network, and 1,792 genes and 2,656 links in the adenoma network. Genes assigned to over-represented biological Gene Ontology terms are highlighted in term specific color. (TIF) [file pone.0086299.s002.tif]

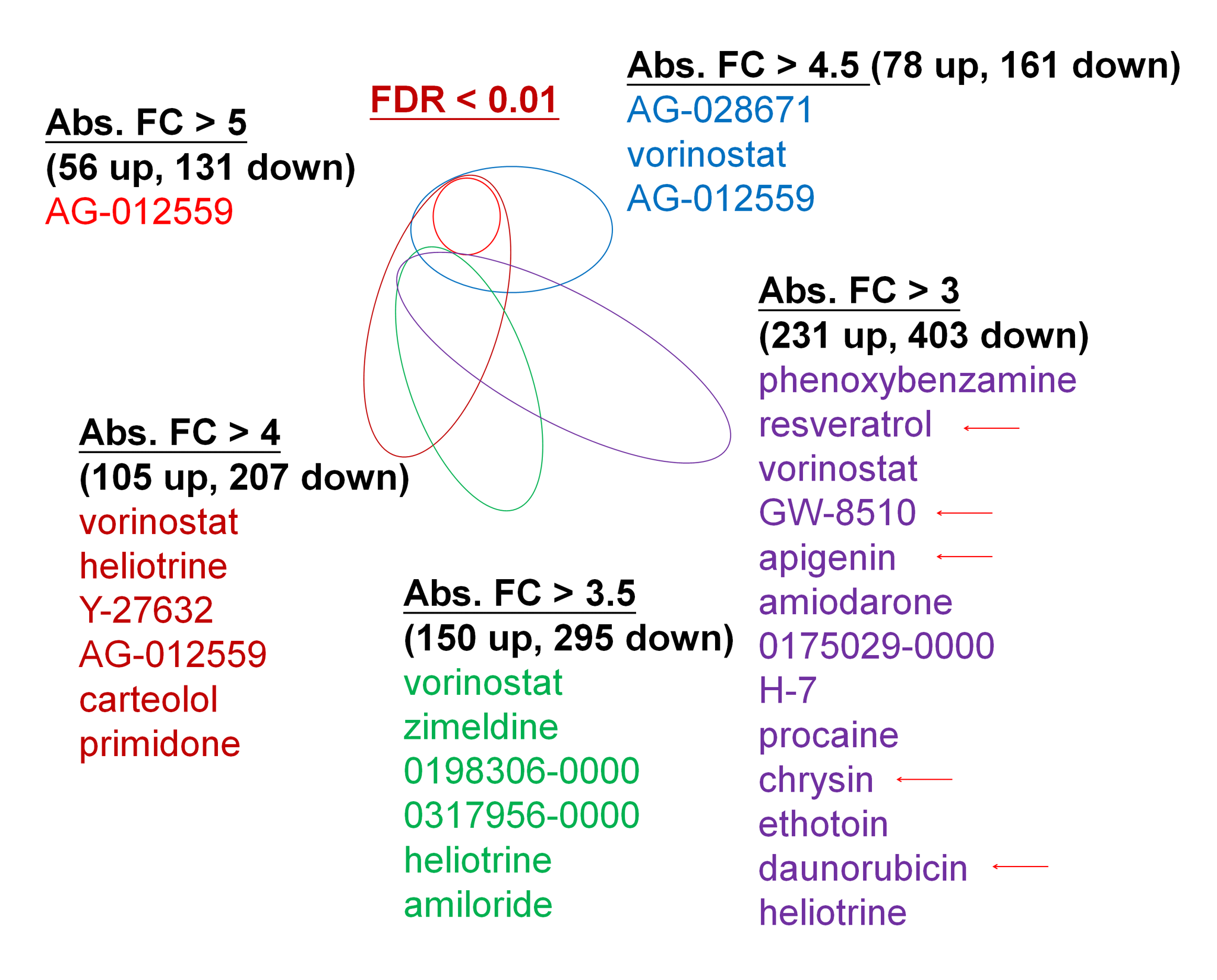

Supplement: Figure S3 — Drugs selected by standard application of CMap, or IGCM, using different fold change (FC) thresholds. Sum of up- and down-regulated genes given under each FC threshold constituted the querying gene set. Drugs listed are those predicted to be beneficial. Red arrow indicates known TTD anti-cancer agents that coincidentally all changed from beneficial at FC = 3 to harmful at FC = 3.5. Vorinostat was the only drug selected at FC >3, 3.5, 4.0, and 4.5; it was also selected in the FMCM procedure. (TIF) [file pone.0086299.s003.tif]

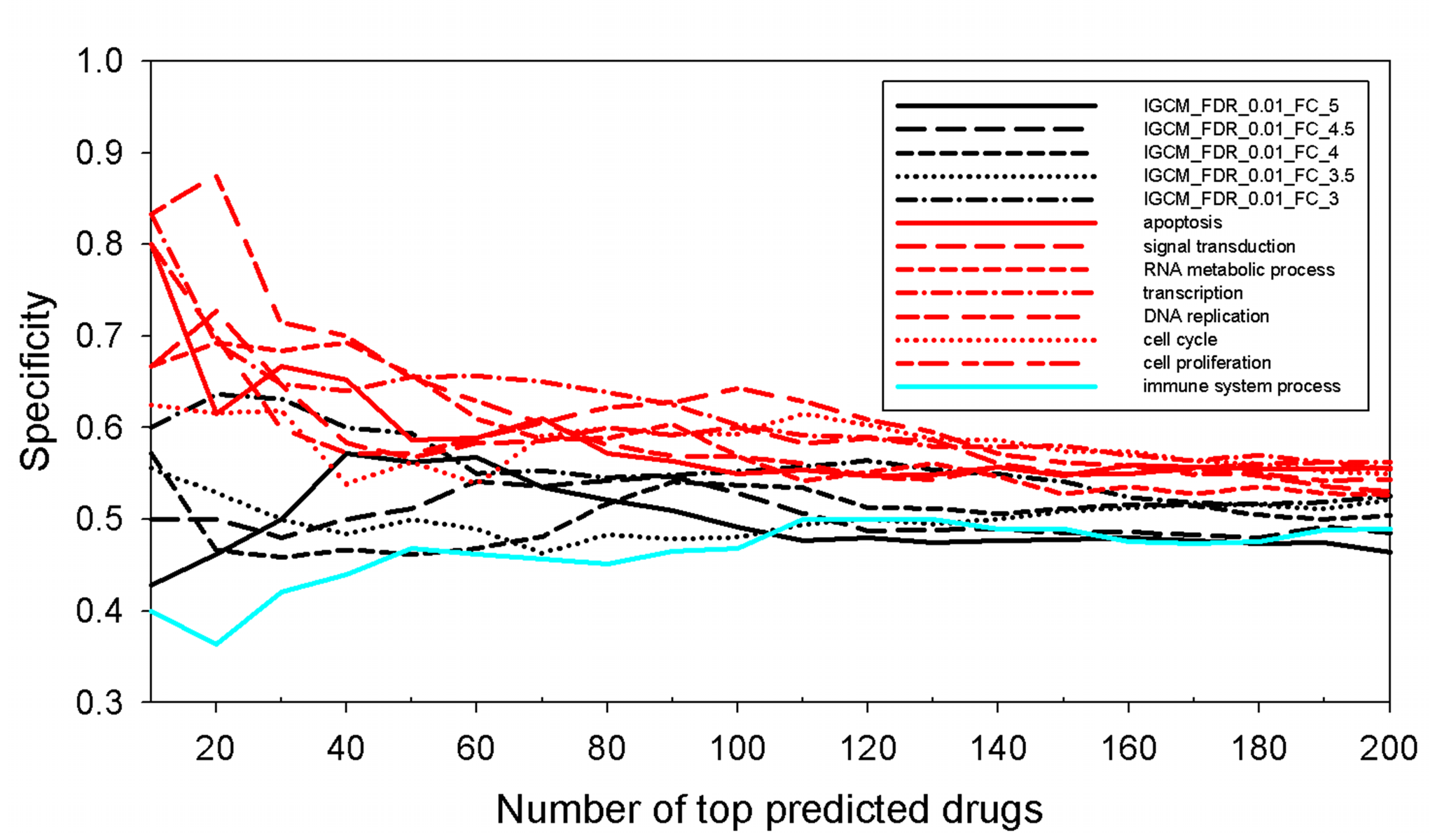

Supplement: Figure S4 — Specificity of predicted drugs. Specificity is true negative (known cancer-inducing agent predicted to be harmful) over all drugs predicted to be harmful; higher specificity implies lower false positive. Seven of the eight FMCM results (red), except immune systems process (cyan), have higher specificities than the five IGCM results (black). (TIF) [file pone.0086299.s004.tif]

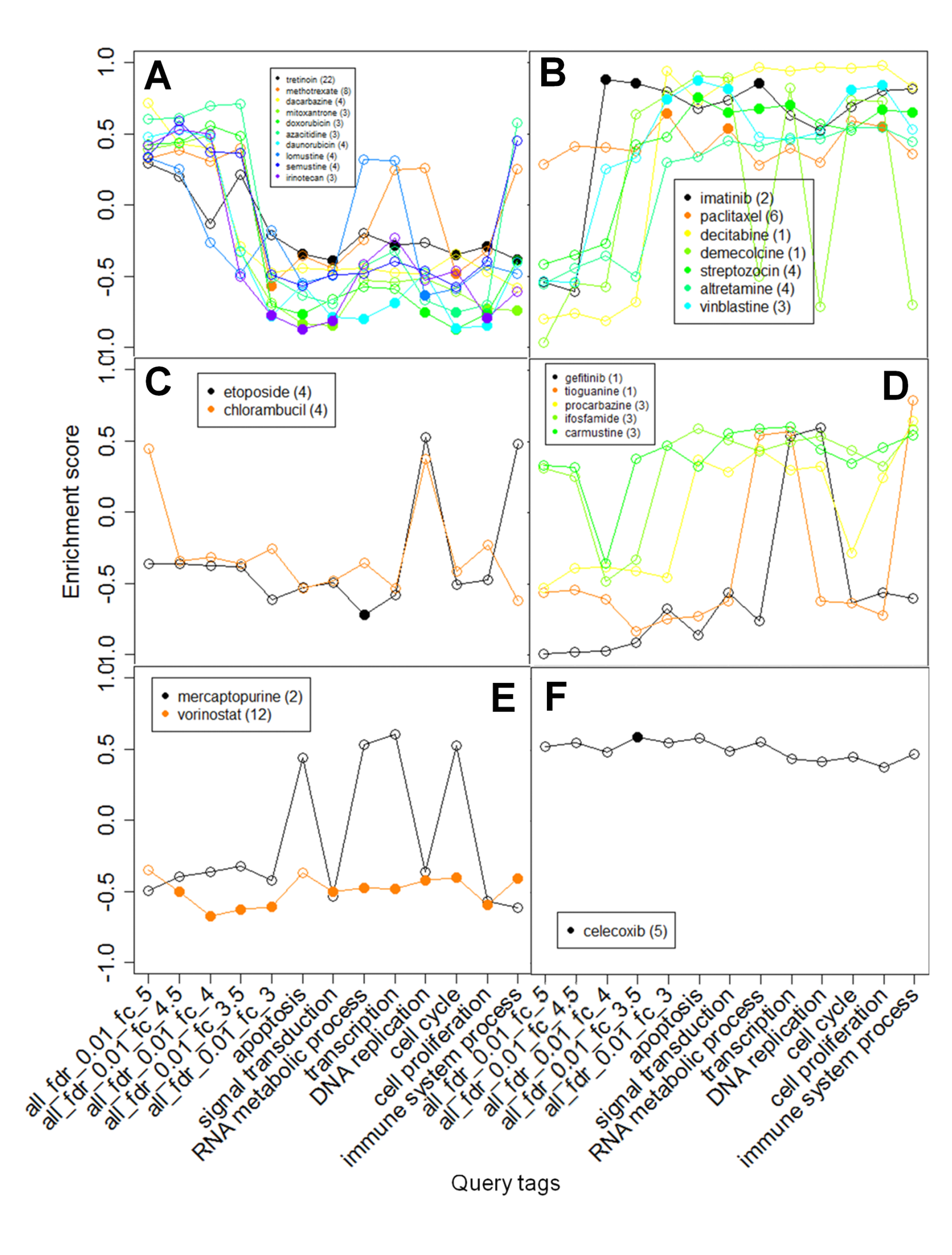

Supplement: Figure S5 — Enrichment scores of 27 chemo-drugs. The 27 chemo-drugs, selected from the L01 class (antineoplastic agents) in the Anatomical Therapeutic Chemical system, are not specific to colon cancer treatment. The ES is those from five IGCM (FC threshold 3 to 5) and eight FMCM runs (FC >0.2). Solid symbol indicates an ES with permutation p value<0.05. The 27 drugs are clustered into six groups according to overall pattern. (TIF) [file pone.0086299.s005.tif]
